# Coupled Hysteresis and Variogram-Controlled Heterogeneity Effects on Underground Hydrogen Storage in Saline Aquifers

**DOI:** 10.1021/acs.energyfuels.6c00987

**Published:** 2026-04-26

**Authors:** Abdolali Mosallanezhad, Amir Jahanbakhsh, Azim Kalantariasl, M. Mercedes Maroto-Valer

**Affiliations:** † Research Centre for Carbon Solutions (RCCS), School of Engineering and Physical Sciences, 3120 Heriot-Watt University, Edinburgh EH14 4AS, U.K.; ‡ Industrial Decarbonisation Research and Innovation Centre (IDRIC), Heriot-Watt University, Edinburgh EH14 4AS, U.K.; § Department of Petroleum Engineering, School of Chemical and Petroleum Engineering, Shiraz University, Shiraz 7134851154, Iran

## Abstract

Hydrogen is projected to be a critical component in worldwide efforts to transition toward low-carbon energy systems. As hydrogen generation capacities rise to meet climate goals, parallel advancements in scalable, high-efficiency storage infrastructure are urgently required. Accurately assessing the storage capacity of saline aquifers is critical for deploying underground hydrogen storage (UHS) on the scale necessary for the energy transition. Conventional assessments, however, often rely on static reservoir properties, such as porosity and permeability, which fail to capture the complex realities of subsurface fluid behavior. This study demonstrates that neglecting crucial dynamic parameters, such as hysteresis, leads to a significant overestimation of recoverable gas. Our numerical simulations reveal that relative permeability hysteresis is one of the dominant factors, causing substantial residual gas trapping that reduces the hydrogen recovery factor by nearly 30% in the initial cycles. The heterogeneous nature of the reservoir adversely affects hydrogen storage operations by promoting preferential flow paths, reducing sweep efficiency, and leading to uneven hydrogen distribution and storage within porous media. Optimal selection of the production rate is crucial for balancing the hydrogen recovery and water management. In heterogeneous models, by optimizing flow rates, recovery improved by ∼10% and water production decreased by ∼20%, underscoring the need for reservoir-specific flow-control strategies. This research underscores the imperative of a dynamic, physics-based approach to designing efficient and cost-effective UHS projects. Moving beyond static assumptions is not just an improvement; it is essential for unlocking the true potential of saline aquifers for large-scale hydrogen storage.

## Graphical abstract

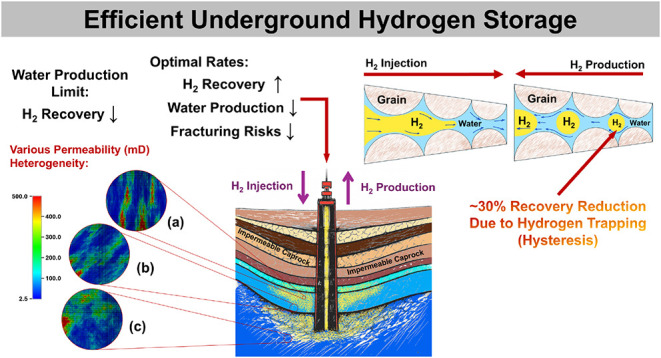

## 1 Introduction

The global consensus to achieve net-zero carbon emissions by 2050, which aims to limit the increase in temperature, underscores the urgency of transitioning to low-carbon energy sources. Over the past few decades, fossil fuels have accounted for a substantial 80% share of the global energy mix, while renewable energy sources have contributed merely 15%. Notably, projections indicate a significant shift, with fossil fuels projected to maintain a 50% share of the energy market. In comparison, renewable energies are expected to triple and hold a 45% share of the energy mix by 2050.^1^

While renewable energy sources, such as solar, wind, and tidal systems, offer significant potential, their inconsistent output, driven by temporal fluctuations, meteorological conditions, seasonal shifts, and regional variability, poses challenges for maintaining stable, dependable energy supplies in large-scale applications.^2^ Hydrogen, characterized by its low density, widespread availability, and substantial energy yield per unit mass, has emerged as a versatile candidate for integrating mobility solutions, electricity production, and combustion-based technologies.^3,4^ Surplus renewable electricity, which might otherwise be grid-curtailed or sold at low value in the market, can be used to power water-splitting electrolyzers, generating hydrogen and oxygen through electrochemical processes.^1,5^ In this context, subsurface hydrogen storage (UHS) has garnered attention as a viable approach for large-scale energy management.^6,7^ Hydrogen can be injected into geological formations for temporary containment and later withdrawal during demand peaks or supply shortages.^8^ Potential geological repositories include depleted fossil fuel reservoirs,^9^ deep saline aquifers,^10^ and salt caverns.^11^

Deep saline aquifers, often composed of porous rocks, such as sandstone, are located at significant depths. They possess considerable capacities worldwide and are geographically widespread, which makes them suitable for interseasonal balancing.^12^ By comparison, depleted hydrocarbon reservoirs offer known structure, existing wells, and established caprock integrity (an advantage for near-term feasibility), yet reactions between liquid hydrocarbons and H_2_ can constrain pure H_2_ storage and complicate gas-quality management.^9,13^ For aquifers, the main trade-offs are the need for robust sealing and pressure management because H_2_ is buoyant and diffusive, and the potential for biogeochemical reactions (e.g., methanogenesis and sulfate reduction to H_2_S) that can alter composition and injectivity. These are the risks that must be assessed and mitigated through site selection and monitoring.^14,15^ Therefore, the choice of storage reservoir location becomes paramount, demanding a stable geological formation that is unlikely to experience such disruptive events.^16^

The successful implementation of underground hydrogen storage (UHS) in aquifers is critically dependent on understanding the complex multiphase flow behavior of hydrogen–brine systems in porous rock, particularly the effects of relative permeability hysteresis and geological heterogeneity.^17–19^ Underground hydrogen storage (UHS) in saline aquifers is cyclic by design (injection, storage, withdrawal), and it raises challenges beyond conventional natural gas storage because of the thermodynamic properties of hydrogen.^14,20^ Stratigraphic layering, permeability anisotropy, and capillary heterogeneity drive uneven plume migration, impact trapping, and shift effective flow properties. Because real aquifers are heterogeneous, even subtle contrasts create preferential pathways and baffles.^21^ Laboratory studies show hydrogen channels through highly permeable areas, bypassing tight layers and leaving unrecovered pockets at the cycle end. Buoyancy-driven flow and viscous fingering of hydrogen are stronger than those of methane due to hydrogen’s lower density and viscosity;^22,23^ classic underground gas-storage work documents fingering under unfavorable mobility ratios,^22^ and recent core-flood imaging shows gravity tongues and capillary-controlled fingering in heterogeneous rock.^24^ The result is poorer sweep and more trapped gas, so geological complexity must be represented explicitly in UHS design.^25^ In short, heterogeneity can both increase trapping (capillary barriers) and cause early breakthrough, making it a first-order uncertainty.

Wettability and contact angle in the rock–brine–hydrogen system control fluid distribution, relative permeability, and residual trapping.^26^ Most evidence indicates that sandstones remain predominantly water-wet during hydrogen injection. According to them, measured hydrogen–brine contact angles on quartz at reservoir conditions are ∼30°–50°;^27,28^ X-ray imaging in Bentheimer reports ∼40°–50° at irreducible water saturation (*S*_wirr_), comparable to methane and nitrogen; Iglauer et al. likewise found sandstone to be strongly water-wet in the presence of hydrogen and brine.^28^ Unlike that for CO_2_, hydrogen rarely shifts rocks away from a water-wet baseline. Still, mineralogy and fluids matter: clays and surface chemistry can raise contact angles and reduce water-wetness (e.g., methane on clay-coated quartz),^27^ and brine composition (including multivalent ions) can modify interfacial behavior.^27^ Field formations with mixed minerals may undergo microbial/chemical alteration, so contact angles vary; overall, hydrogen behaves as the nonwetting phase, occupying larger pores and being residually trapped in smaller ones during imbibition.

Relative permeability (*k*_r_) quantifies hydrogen–brine multiphase flow as a function of saturation and depends on the path; drainage during injection versus imbibition during withdrawal, while coupling to capillary pressure.^19,29^ Robust hydrogen *k*_r_ data remain limited. Early core floods by Yekta et al. reported very low hydrogen mobility (gas end point *k*_r_ ≈ 0.04 at 60% gas saturation during drainage), likely influenced by hydrogen’s low viscosity and capillary-end effects, raising concerns about injectivity and recovery.^30^ To bridge narrow saturation windows, they analytically extended *k*_r_ using measured capillary pressure data, underscoring the need for consistency between Pc and kr. Higher entry pressures reduce gas connectivity and thus *k*_r_ at a given saturation.^26,31^ Their results suggested that hydrogen’s viscosity remains nearly constant under storage conditions (<100 bar, < 100 °C), implying minimal impact on capillary number and relative permeability in the hydrogen–water system.^30^ In another study conducted by Rezaei et al., an unsteady-state experimental approach was employed to analyze relative permeability trends in hydrogen-water systems under varying conditions such as temperature, pressure, water salinity, and rock properties.^32^ Their work also contrasted hydrogen’s flow behavior with methane (CH_4_) and nitrogen (N_2_). The findings highlighted complex pressure-dependent interactions: higher pressures diminished hydrogen’s relative permeability at elevated gas saturation levels, linked to increased gas viscosity. Conversely, rising pressure enhanced permeability under low gas saturation as capillary forces outweighed viscous effects. Alterations in water salinity exhibited negligible influence on hydrogen’s permeability trends. When evaluating rock characteristics, porosity was identified as the dominant factor shaping permeability curves, with minimal correlation to pore geometry or mineralogy. Comparative analysis showed that CH_4_ maintained higher permeability than H_2_ and N_2_ across most saturation ranges. However, at maximum saturation, H_2_ and N_2_ outperformed CH_4_, a phenomenon attributed to hydrogen’s reduced viscosity.^22,32^ This work underscores the critical role of fluid dynamics and rock structure in hydrogen transport within subsurface environments.

Hysteresis in relative permeability under cyclic injection/production is therefore pivotal. Because trapped hydrogen becomes disconnected during imbibition, the gas relative permeability on imbibition is lower than on drainage; water connectivity also recovers differently. The first hydrogen–water hysteresis measurements across primary drainage, imbibition, and secondary drainage (Lysyy et al.) showed pronounced hysteresis: at the same intermediate gas saturation, imbibition *k*_r_ was markedly lower, and reinjection did not fully restore initial mobility; residual trapping reduced the effective gas saturation for subsequent cycles.^18,33^ Bo et al. showed that omitting gas-phase hysteresis can overestimate cumulative hydrogen production by up to 338%, while using analogue natural gas *k*_r_ can underestimate producible hydrogen by ∼141%. Consistent with conventional gas-storage literature, hysteresis broadens the gas–water mixing zone and lowers working-gas capacity if neglected.^17^

Researchers have also increasingly relied on pore network modeling (PNM) to gain deeper insights into the relative permeability of hydrogen-water systems. PNM employs a network of pores that is topologically and geometrically analogous to a rock sample to predict capillary pressure and relative permeability by simulating fluid flow through the network’s elements.^34^ Hashemi et al. investigated the relative permeability and capillary pressure of the hydrogen-water system with the help of PNM. Through their simulations, they utilized Corey functions to model relative permeability, with exponents of 1.3 for hydrogen and 4.4 for water. These values reflect the dominant wetting phase (water) and are consistent with expected pore geometry and wettability characteristics in sandstone reservoirs.^34^ Moreover, CFD-based direct numerical simulations have shown that hydrogen–water relative permeability can depend on capillary number and pore-scale heterogeneity, and that hysteresis effects may be stronger in more complex geometries.^35^ These advances motivate the need for a Darcy-scale framework that preserves hydrogen-specific hysteresis while enabling controlled comparisons across heterogeneous architectures, particularly the directional connectivity that governs preferential pathways and bypassing in aquifers.

To date, many UHS simulations have relied on analogous gas–water flow functions and have often simplified the roles of saturation history and geological heterogeneities. Our study seeks to bridge this gap by developing a specialized, hydrogen-focused modeling framework that connects the observation and outcome of core-scale experiments with Darcy-scale simulations. A key innovation is how we integrate hysteresis in hydrogen–brine flow functions with controlled, statistically comparable heterogeneity realizations, allowing us to assess the influence of geological variations without confounding factors. In this work, we therefore couple laboratory-derived hydrogen–brine relative permeability and capillary pressure hysteresis, implemented using the Modified Killough formulation with Leverett J-function scaling, with four statistically matched absolute permeability realizations generated using the Sequential Gaussian Simulation (SGS) method. In addition, by smoothing abrupt transitions in relative permeability, we decrease numerical dispersion and mitigate convergence challenges that often occur when simulating high-mobility gas flow in porous media, particularly on larger scales. One realization is omni-directional, and three are anisotropic, with principal continuity directions aligned at 0°, 45°, and 90° relative to the injection direction. Crucially, all four realizations are designed to share the same average (mean) permeability, variance, and search-neighbor correlation area, ensuring comparability across cases. This variogram-controlled SGS design is a key innovation of the study, and it enables a clean assessment of how heterogeneity orientation (not just heterogeneity magnitude) interacts with hysteresis to alter preferential pathways, breakthrough timing, water influx, pressure dynamics, and ultimately gas recovery. Finally, building on these insights, we evaluate flow rate strategies tailored to each geological heterogeneity architecture under operational constraints, providing practical guidance for early-stage UHS design in saline aquifers.

## 2 Methodology

### 2.1 Dynamic Model Development

This section outlines the primary steps for developing the two-phase flow model in the CMG-GEM simulator. The focus here is to examine the influence of hydrogen trapping on injection and production cycles in porous media. In this context, the model primarily incorporates hysteresis effects within the Darcy flow equations. The following equations account for water–gas relative permeability hysteresis and capillary pressure hysteresis effects. In these equations, the superscripts “Im,” “Dr,” and “Scan” correspond to the imbibition, drainage, and scanning phases, respectively.

#### 2.1.1 Hysteresis Model

The gas trapping phenomenon was simulated by employing Land’s correlation. Equation 1 represents Land’s equation within the water–gas system.^36^

1 $$S_{g_{\mathrm{rh}}}=S_{g_{\mathrm{crit}}}+\frac{1}{\frac{1}{S_{gr_{\max}}}-\frac{1}{1-S_{w_{\mathrm{con}}}-S_{g_{\mathrm{crit}}}}+\frac{1}{S_{g_{\mathrm{hy}}}-S_{g_{\mathrm{crit}}}}}$$

where *S*_g_rh__ denotes the residual gas saturation during water imbibition, *S*_g_hy__ is the point at which the drainage to imbibition reversal occurs, representing the highest achieved gas saturation, *S*_g_crit__ stands for the critical gas saturation, and *S*_grmax_ signifies the maximum residual gas saturation, a parameter that requires determination through the imbibition water–gas capillary pressure (*P*_cgw_i__) curve.

#### 2.1.2 Relative Permeability Model

For modeling relative permeability hysteresis, two approaches are commonly used: Carlson’s equations^37^ and Killough’s method.^38^ In cases where the imbibition curve is not explicitly defined, Carlson’s eq (eqs 2 and 3) is employed as the default procedure.

2 $$K_{\mathrm{rg}}^{\mathrm{scan}}\left(S_{g}\right)=K_{\mathrm{rg}}^{\mathrm{Dr}}\left(S_{g}\left(\mathrm{shifted}\right)\right)$$

Here, *S*_g_ (shifted) is defined as

3 $$S_{g}\left(\mathrm{shifted}\right)=S_{g_{\mathrm{crit}}}+\left(S_{g}-S_{g_{\mathrm{rh}}}\right)\frac{S_{g_{\mathrm{hy}}}-S_{g_{\mathrm{crit}}}}{S_{g_{\mathrm{hy}}}-S_{g_{\mathrm{rh}}}}$$

The Killough relative permeability interpolation method was used in this study. Equations 3 and 4 are employed, and eq 5 can be utilized for the Killough saturation interpolation method.

Killough’s relative permeability interpolation

4 Krgscan(Sg)=KrgIm(Sgnorm)KrgDr(Sghy)KrgDr(1−Swcon)

Here normalized gas saturation (*S*_g_^norm^) is estimated by

5 $$S_{g}^{\mathrm{norm}}=S_{gr_{\max}}+\frac{\left(S_{g}-S_{g_{\mathrm{rh}}}\right)\left(1-S_{w_{\mathrm{con}}}-S_{gr_{\max}}\right)}{S_{g_{\mathrm{hy}}}-S_{g_{\mathrm{rh}}}}$$

Killough’s saturation interpolation

6 $$K_{\mathrm{rg}}^{\mathrm{scan}}\left(S_{g}\right)=K_{\mathrm{rg}}^{\mathrm{Dr}}\left(S_{g_{\mathrm{hy}}}\right){\left(\frac{S_{g}-S_{g_{\mathrm{rh}}}}{S_{g_{\mathrm{hy}}}-S_{g_{\mathrm{rh}}}}\right)}^{\in }$$

where the exponent ∈ is defined by the user.

Killough’s is an interpolative scheme that constructs scanning curves between measured drainage and imbibition bounds, thereby directly incorporating all available experimental evidence. By contrast, Carlson’s method is predictive, deriving imbibition behavior from the drainage curve plus a trapping model (e.g., Land), and is most appropriate when imbibition data are unavailable. As a result, we chose Killough’s hysteresis formulation because the data set includes hydrogen–water core-flood measurements with both primary drainage and secondary imbibition curves.^37,38^

#### 2.1.3 Capillary Pressure Model

Incorporating capillary pressure hysteresis, the water–gas capillary pressure (*P*_cgw_) can be expressed as described here. When the water saturation begins to increase on the drainage curve, the water–gas capillary pressure is determined from the drainage-to-imbibition scanning curve

7 Pcgw(Sw)=f×Pcgw(SwIm)+(1−f)×Pcgw(SwDr)

The factor *f* lies between zero and one and is given by

8 $$f=\left(\frac{S_{w_{\left(\max\right)}}-S_{w_{\left(\mathrm{hyst}\right)}}+\varepsilon }{S_{w_{\left(\max\right)}}-S_{w_{\left(\mathrm{hyst}\right)}}}\right)\left(\frac{S_{w}-S_{w_{\left(\mathrm{hyst}\right)}}}{S_{w}-S_{w_{\left(\mathrm{hyst}\right)}}+\varepsilon }\right)$$

where *S*_w_(hyst)__ is the water saturation when the reversal happened and *S*_w_(max)__ is the maximum water saturation in porous media (1-*S*_grw_). Before water saturation falls below *S*_w_(hyst)__, *P*_cgw_ continues to exist on the scanning curve. If the water saturation starts to decrease during imbibition, the water–gas capillary pressure is determined using the imbibition-to-drainage scanning curve

9 Pcgw(Sw)=f×Pcgw(SwDr)+(1−f)×Pcgw(SwIm)

where

10 $$f=\left(\frac{S_{w_{\left(\mathrm{hyst}\right)}}-S_{\mathrm{wr}}+\varepsilon }{S_{w_{\left(\mathrm{hyst}\right)}}-S_{\mathrm{wr}}}\right)\left(\frac{S_{w_{\left(\mathrm{hyst}\right)}}-S_{w}}{S_{w_{\left(\mathrm{hyst}\right)}}-S_{w}+\varepsilon }\right)$$

with *S*_wr_, the connate water saturation, and ε is the value based on experimental data. Before water saturation exceeds *S*_w_(hyst)__, *P*_cgw_ remains on the scanning curve.

Since absolute permeability heterogeneity is defined to capture hydrogen storage efficiency, the J-function approach can be utilized to adjust the capillary pressure reflecting variations in porosity and permeability across different reservoir blocks. When using the Gravity Equilibrium initialization method, the influence of variations in porosity, permeability, and surface tension is incorporated, leading to spatial differences in transition zones. These variations arise due to changes in the capillary pressure throughout the reservoir. Relative permeability is expected to depend on interfacial tension and operating conditions; however, hysteresis-consistent hydrogen–brine data sets with both drainage and imbibition curves across multiple pressures and temperatures remain scarce. Since our hysteresis workflow depends on bounded drainage and imbibition curves, adding IFT-dependent relative permeability without matching imbibition data would result in insufficient constraints. We therefore fixed the hysteretic flow functions to the best available experimental data set and prioritized condition-dependent drainage/imbibition measurements for future calibration.

11 $$J_{c_{\left(S_{w}\right)}}=\frac{P_{\mathrm{cgw}}}{\sigma \times \sqrt{\frac{\phi }{K}}\times 31.8316}$$

where *P*_cgw_ is the water–gas capillary pressure in kPa, *J*_c(S_w_)_ is a dimensionless J-function, ϕ is the grid block porosity, *K* is the block absolute permeability (mD), and σ is the gas–water surface tension × Cosine (Contact Angle) (dyn/cm).

Three simulations were conducted to understand hydrogen trapping during the injection and production cycles. The first simulation involved a one-dimensional two-phase flow analysis constructed using the CMG-GEM simulator. Rather than directly adopting reported curves from studies such as Yekta et al.,^30^ Hashemi et al.,^34^ and Lysyy et al.,^33^ the one-dimensional simulation was developed to generate smooth and physically consistent relative permeability and capillary pressure curves for hydrogen–brine flow, incorporating hysteresis effects.

We constructed a simplified 1D model to validate against experimental data. The other reason was to have faster runtimes and better control over the flow function behavior. This was necessary to avoid instability and poor numerical convergence caused by sharp transitions in the reported curves. The experimental data on relative permeability for primary drainage (PD) and secondary imbibition (SI) provided by Lysyy et al. were employed to construct the relative permeability and capillary pressure curves, incorporating gas hysteresis effects (Figure 1). The relative permeability and capillary curves are represented using an LET formulation provided by Lysyy et al., which is then used within the hysteresis workflow.^33^

**Figure 1:**
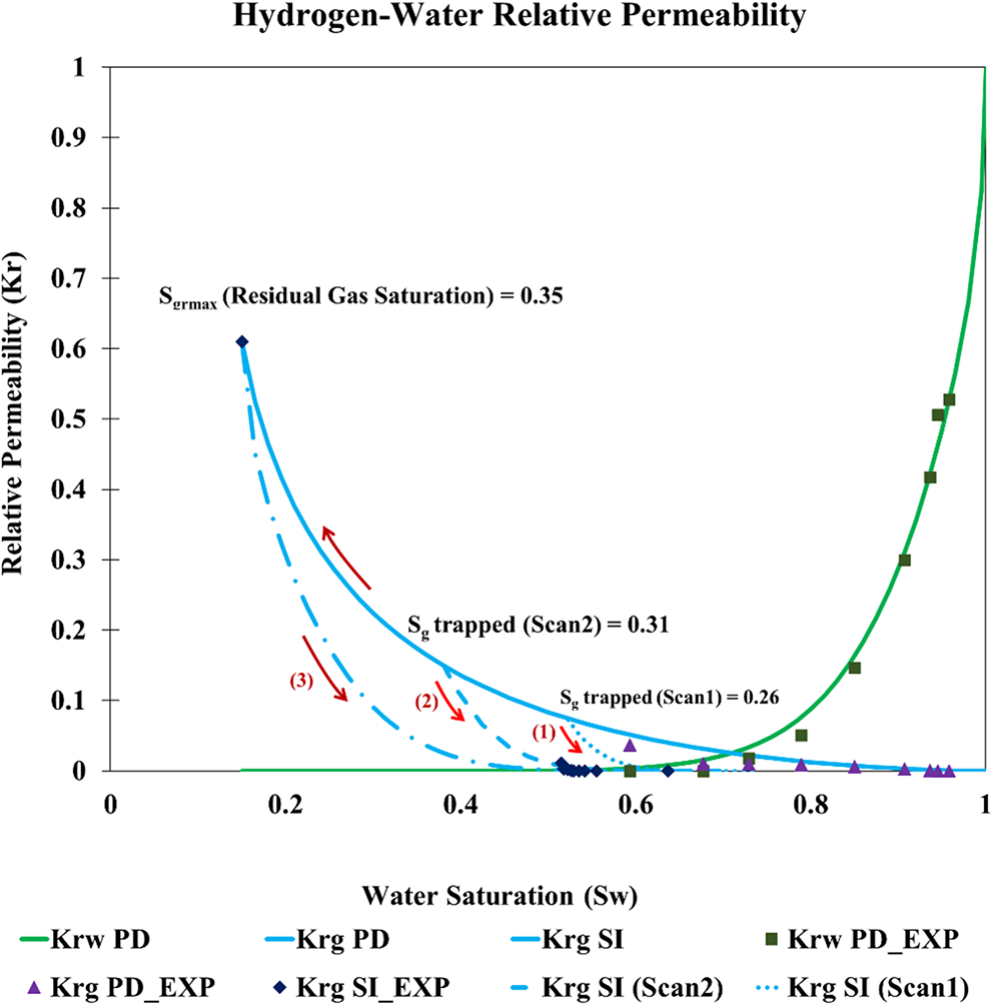
Hydrogen–water relative permeability curves showing pronounced hysteresis between primary drainage (PD) and secondary imbibition (SI).^33^ Early cycle reversal before irreducible water saturation follows scanning curves (Scan 1 and 2), which are interpolated between the bounding PD and SI curves.

Much of the available literature derives drainage *k*_r_ from unsteady-state core floods with history matching rather than dense steady-state sampling, producing limited discrete saturation points;^22,32^ other data sets focus on drainage only and do not provide imbibition data.^30^ Lysyy et al.’s study was selected because it provides both drainage and imbibition information, enabling a hysteresis-consistent scanning-curve construction with better overall coverage than alternative data sets currently available.

1D simulations allow us to approximate missing hysteresis effects and refine the relative permeability and capillary pressure curves for more representative reservoir-scale modeling. The maximum residual gas saturation was fixed at 0.35, which agrees with their results. Meanwhile, the critical gas saturation values were adjusted based on findings from Hashemi et al. and Rezaei et al. that involved comparable core characteristics (Figure 1). The scanning curve was optimized using Killough’s relative permeability and saturation interpolation method (eqs 3–5), providing a smooth transition between drainage and imbibition cycles. This approach ensures a more accurate representation of hysteresis effects in a multiphase flow. The optimization was based on the secondary drainage input parameters reported by Lysyy et al., ensuring consistency with experimentally derived relative permeability trends. The gas relative permeability (*k*_rg_) shows significant hysteresis, meaning krg during secondary imbibition is lower than during primary drainage, particularly at higher water saturation. This decrease in *k*_rg_ highlights diminished hydrogen mobility in subsequent cycles as a result of immobile gas saturation in the pores. At the same time, the water relative permeability *k*_rw_ remains suppressed because the system wettability remains strongly water-wet.

Figure 1 also illustrates early cycle reversal and the resulting scanning behavior. During fluid displacement, if a grid block reverses from drainage to imbibition before reaching irreducible water saturation, then it follows a scanning curve interpolated between the bounding primary drainage and secondary imbibition curves (Killough interpolation). As a result, some gas becomes disconnected and gets trapped. The trapped gas saturation *S*_gtrapped_ at the end of that partial imbibition is lower than the end point residual gas saturation *S*_grmax_. In the figure, the trapped gas saturations of the first and second early reversal cycles are 0.26 and 0.31, respectively (Figure 1: Curves 1 and 2).

The Leverett J-function for capillary pressure (Figure 2) shows a distinct separation between the primary drainage (PD) and secondary imbibition (SI) curves, forming a hysteresis loop. During primary drainage, the J-function increases sharply at low water saturation (*S*_w_ ≈ 0.2), reflecting the high capillary pressure required for hydrogen to displace brine in the water-wet sandstone.

**Figure 2:**
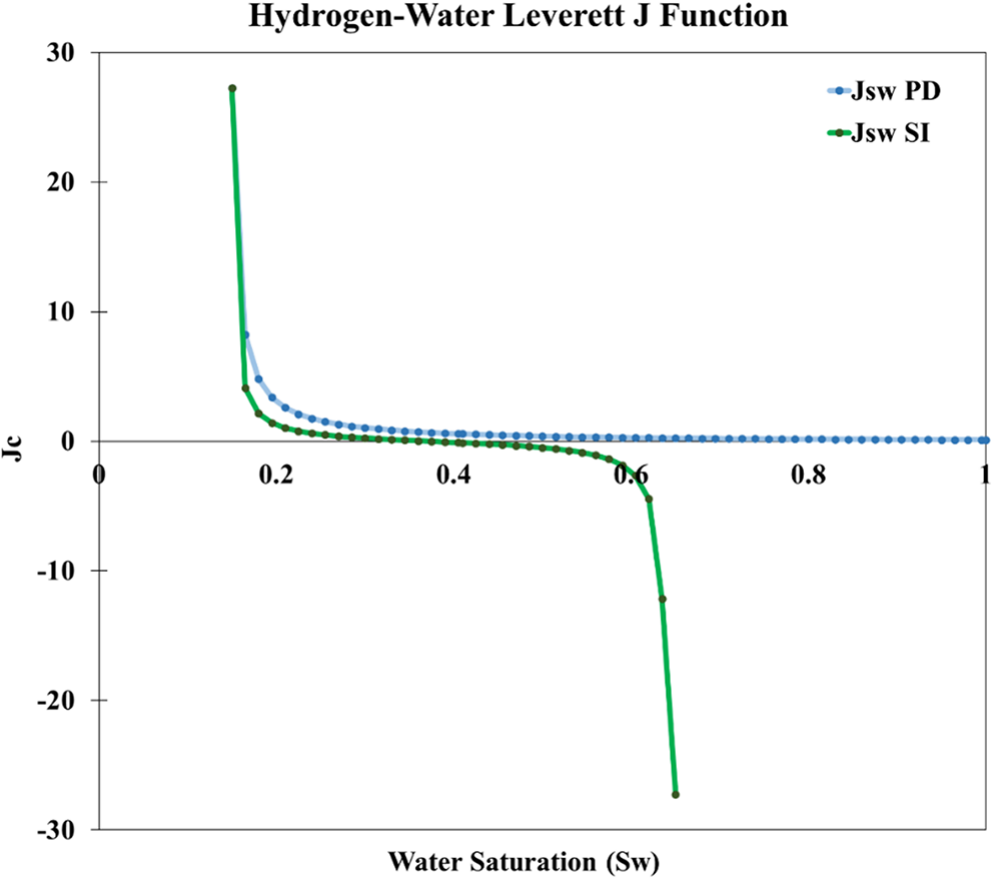
Leverett J-function of primary drainage (PD) and secondary imbibition (SI) (calculated using capillary pressure correlation provided in Lysyy et al.^33^).

Subsequently, two-dimensional two-phase flow models were developed to explore the impact of relative permeability hysteresis on the injection and production cycles. It is important to note that scaling up from 1D core-scale validation to 2D large-scale simulation introduces uncertainties. The 1D model ensured that the flow functions replicated laboratory experiments (which are principally 3D flows), assuming that gravity segregation did not occur in the core-flood experiments. When moving to 2D, additional factors such as spatial heterogeneity, well geometry, and multidirectional flow come into play. Some discrepancies are inevitable because the 2D and 1D models cannot be perfectly congruent (e.g., 2D grid discretization can reintroduce numerical dispersion that was negligible in 1D). In the next step, we performed a grid refinement check using 10 × 80, 50 × 400, and 100 × 800 areal grids and the residual gas saturation sensitivity analysis was performed using the Carlson’s correlation to vary residual trapping behavior; this sensitivity is reported as a bounded test of *S*_grmax_ uncertainty, while the base-case cyclic results use Modified Killough scanning curves. Nevertheless, the lack of field-scale data for direct calibration means that our 2D results assume that core-scale parameters remain valid at larger scales.

Prior to our main simulations, we conducted additional sensitivity analyses of aquifer boundary conditions and cycle stabilization.

The final set of simulations examined the impact of injection and production rates as well as system heterogeneity on hydrogen storage performance. The models were designed based on experimental and numerical studies of sandstone. The relevant properties of the porous media and the fluids employed are detailed in Table 1. Hydrogen properties are computed with the Peng–Robinson EOS; the following values are at 10 MPa and 45 °C, while viscosity and density are updated dynamically as pressure evolves during cycling.

**Table 1:** Rock and Fluid Properties for the Hydrogen-Brine Numerical Study

formation	rock		fluid (10 MPa, 45 °C)			
Sandstone	Porosity (%)	Permeability (mD)		Interfacial Tension (mN/m)	Density (kg/m^3^)	Viscosity (cP)
	18.33	100	Hydrogen	46	7.2	0.00954
			Brine		994.5	0.59

### 2.2 Homogeneous Model Development

In the proposed homogeneous model for two-dimensional two-phase flow, a series of two-dimensional horizontal models was constructed to examine the residual effects on the hydrogen storage performance on a larger scale. These models continued to utilize relative permeability and capillary pressure curves obtained from the previous phase of the study. This study targets short, injection–withdrawal cycles in which viscous force and hysteretic *k*_r_–Pc dominate plume evolution and gas recovery. Over these time scales, molecular diffusion is expected to be minor at the model scale and thus far smaller than the grid spacing and the domain length scales controlling migration. Hydrogen dissolution and geochemical/biogeochemical reactions can become important over longer storage and rest periods by transferring H_2_ into the aqueous phase or altering composition and injectivity; these processes are therefore treated as longer-time scale loss mechanisms and are explicitly left for coupled reactive-transport extensions (see Section 5).

The final simulations were run in a two-dimensional Cartesian system consisting of 100 × 800 × 1 blocks, as depicted in Figure 3. The dimensions and physical properties of the models used for simulation are provided in Table 2. The simulation model was designed to align horizontally, resembling the layout of the experimental floods. An Injection/production well was configured (at the right side of the reservoir) to operate at a fixed rate of 6.15 m^3^/day under reservoir conditions. This rate was chosen to prevent formation fracturing after 30 days of hydrogen injection. The cycling well was placed to impose a controlled displacement that defines a single injection direction and enables a clean comparison of variogram orientation effects, which will be added to the model in the next steps. Therefore, the results should be interpreted as areal-sweep behavior under controlled displacement rather than as a site-specific well-pattern prediction.

**Figure 3:**
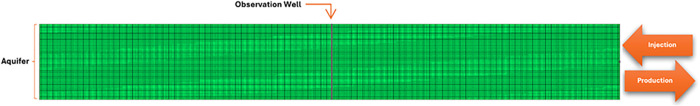
Homogeneous two-dimensional model of the water–hydrogen system.

**Table 2:** Specifications of the Two-Dimensional Two-Phase Flow Model

grids	size of grids (m)	length (m)	width (m)	initial pore volume (m^3^)	gas injection rate (m^3^/day)	production rate (m^3^/day)
100 × 800 × 1	Δ*x* = 1	800	100	7332	6.15	6.15
	Δ*y* = 1					
	Δ*z* = 0.5					

To analyze the sensitivity of the models to boundary conditions, the model was configured to simulate three types of aquifers: closed, semiclosed, and open. The closed aquifer has no effective external boundary, the semiclosed aquifer is characterized by limited hydraulic conductivity, and the open aquifer has a constant-pressure boundary. After the sensitivity analysis, it was decided to select an open aquifer as our main boundary condition (See Supporting Information, Figures S4 and S5).

To establish a constant-pressure boundary, the aquifer was connected to the grid edge along the model boundary (*i* = 1:100, *j* = 800, and *k* = 1). The initial pressure in the aquifer was set to 10,000 kPa, referenced at a datum depth of 1500.25 m. Water exchange was modeled using the constant-pressure formulation, where inflow and outflow depend on the pressure difference between the aquifer boundary pressure and the reservoir pressure, with the effective hydraulic conductivity governed by aquifer properties (thickness = 100 m, porosity = 0.1833, permeability = 100 mD). Leakage was activated to allow communication in both directions when the reservoir pressure exceeds the aquifer pressure, ensuring the boundary functions as a pressure-support proxy rather than a one-way source. The geometry option is rectangular and infinite, which does not affect the calculation of the hydraulic influx.

A straightforward operational strategy was devised to investigate the impact of residual effects on the efficiency of hydrogen injection operations. The model was simulated under two conditions: one that did not account for gas trapping and another that accounted for hysteresis. Subsequently, the hydrogen storage performance was compared. Operational parameters for periodic hydrogen injection and production phases are outlined in Table 3. The simulation incorporated a fixed-pressure boundary condition, representing a large-scale aquifer system, to enable continuous gas migration and maintain uniform flow dynamics during successive operational phases. In all scenarios, a dedicated monitoring well located at the reservoir’s geometric center tracked real-time changes in hydrogen saturation. Injection protocols mandated termination upon hydrogen detection at this location to prevent unintended gas displacement, with operations resuming only in subsequent cycles. Injection BHP was capped at 20 MPa as a conservative screening constraint to avoid unrealistically high pressurization in a fully water-saturated model. Because fracture pressure is controlled by the in situ stress state and rock tensile strength, the exact cap is site-specific; here, it is used as a representative upper bound for sensitivity-consistent comparisons. Also, production phases maintained a minimum wellbore pressure of 5000 kPa to ensure stable extraction efficiency.

**Table 3:** Operational Strategy of Hydrogen Storage Using the Homogeneous Model

operational strategy	first injection (0–30 D)	first production (30–60 D)	second injection (60–90 D)	second production (90–120 D)
	H_2_ Injection	Production	H_2_ Injection	Production
	6.15 m^3^/day	6.15 m^3^/day	6.15 m^3^/day	6.15 m^3^/day

### 2.3 Heterogeneous Model Development

To investigate how geological heterogeneity architecture affects UHS performance under hysteretic flow, we generated four absolute permeability realizations using the Sequential Gaussian Simulation (SGS) method.^39^ SGS was selected because it produces geologically plausible spatial variability while allowing precise control over the target distribution (log-normal permeability), mean and variance, and spatial continuity, as described by a variogram model. The design objective was comparability: all realizations share the same grid, permeability range, log-normal marginal distribution, and target mean permeability (100 mD), while differing only in the direction of spatial continuity, which potentially represents different geological settings (Figure 4). Note that porosity was held constant (ϕ = 0.1833) across all models to isolate the effect of permeability connectivity architecture on the cyclic UHS performance. The SGS workflow was applied only to the permeability. Porosity–permeability cosimulation using formation-specific ϕ–k relationships is therefore left as a site-specific extension for future work.

**Figure 4:**
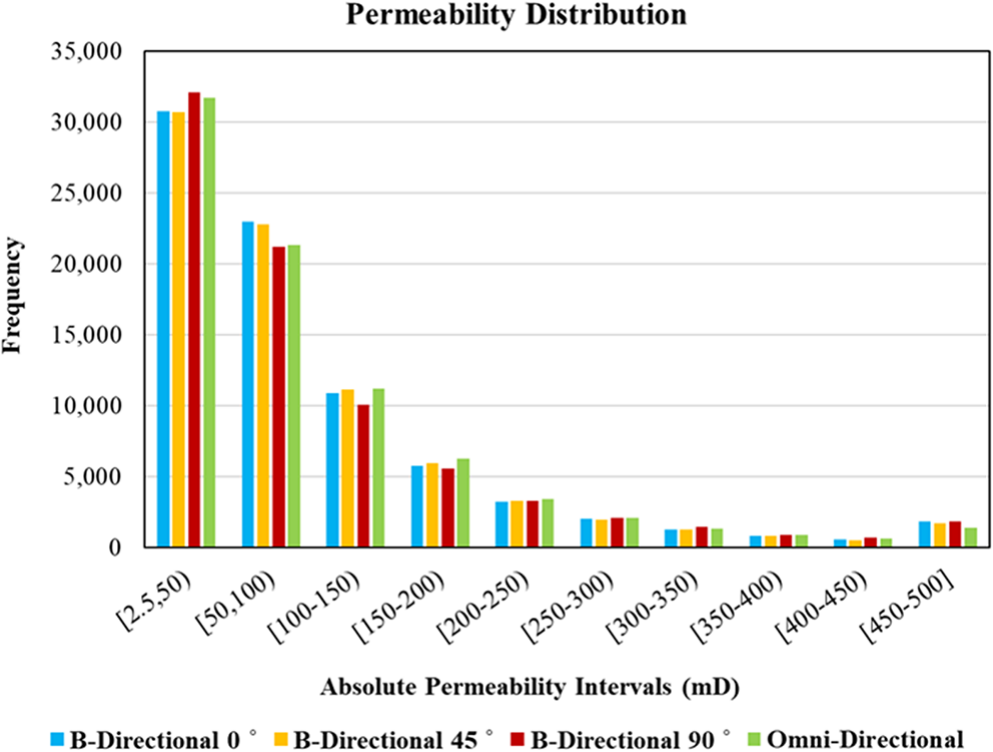
Permeability distribution in the four generated maps.

To isolate the impact of heterogeneity orientation while keeping the statistical structure comparable across realizations, we generated four SGS absolute permeability fields using a consistent exponential variogram family with identical nugget and sill (C0 = 0.05, C1 = 0.95) and a fixed effective correlation/support area. Realization (a) is isotropic (omni-directional) with equal ranges (circular search area). Realizations (b–d) introduce geometric anisotropy with a shared major/minor range pair (elliptic search area) while rotating the principal continuity direction to 0°, 45°, and 90° relative to the injection direction. The anisotropic cases were designed to preserve an equal-area neighborhood, meaning all four realizations share the same correlation and differ only in the continuity orientation. Because the smallest range (42.7 m) remains larger than the grid block size, heterogeneity is spatially resolved rather than numerically averaged, enabling a comparable assessment of how connectivity orientation controls preferential flow, breakthrough, trapping, and water production. The full variogram specifications are summarized in Table 4. Moreover, for each variogram class, more than 50 realizations were generated, and the statistically closest realizations (matched mean permeability and most similar permeability distribution) were selected to ensure like-for-like comparability across orientations.

**Table 4:** Variogram Specifications of the Heterogeneous Models

realization	continuity type	variogram model	nugget	sill	major range (m)	minor range (m)	azimuth (°)
a	Omni-Directional	Exponential	0.05	0.95	80	80	N/A
b	Bidirectional	Exponential	0.05	0.95	150	42.7	0
c	Bidirectional	Exponential	0.05	0.95	150	42.7	45
d	Bidirectional	Exponential	0.05	0.95	150	42.7	90

All SGS realizations were generated on the same 2D domain and then used directly as permeability input for the hysteretic UHS simulations. By holding distributional statistics and correlation area constant while rotating the principal continuity direction, the workflow isolates the effect of heterogeneity orientation on preferential pathways, breakthrough timing, pressure behavior, and water influx during cyclic injection/production.

The absolute permeability map is illustrated in Figure 5a–d. It is noteworthy that hysteresis was accounted for in all of these models. For the geological mapping, a range of absolute permeability values from 2.5 to 500 mD was selected. In the first heterogeneous model, spatial continuity was established as omni-directional, and the absolute permeability was estimated at unspecified points within circular regions. Figure 5a provides an overview of the absolute permeability map for this model, generated through the SGS simulation method. In the second heterogeneous model, spatial continuity was designated as Bidirectional, with absolute permeability estimated at unspecified points within elliptical regions oriented at 0° relative to the fluid flow direction (Figure 5b). The third heterogeneous model also featured Bidirectional spatial continuity, with absolute permeability estimated at unspecified points within elliptical regions, at an angle of 45° relative to the fluid flow direction (Figure 5c). Lastly, the fourth heterogeneous model implemented an absolute permeability estimation strategy using elliptical regions oriented at 90° to the fluid flow axis (Figure 5d).

**Figure 5:**
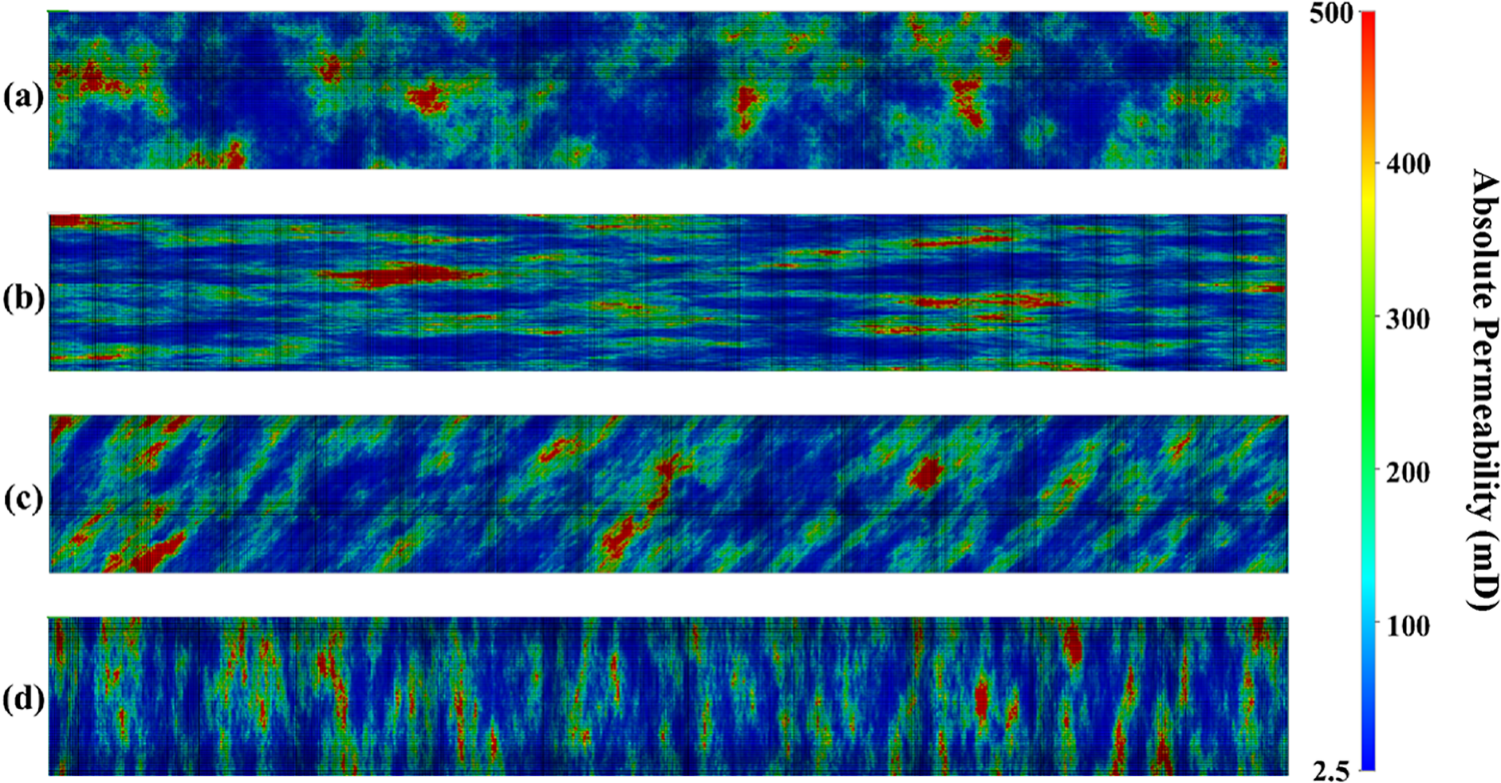
Absolute permeability map generated by the SGS simulation method. (a) Omni-Directional continuity, (b) bidirectional continuity with an angle of 0°, (c) bidirectional continuity with an angle of 45°, and (d) bidirectional continuity with an angle of 90°.

Before simulating hydrogen storage in four generated porous media and having a better understanding of fluid flow dynamics in the four permeability distribution scenarios (0°, 45°, 90° bidirectional, and omni-directional anisotropy), a study of flow direction and breakthrough time was conducted. Since permeability anisotropy significantly influences fluid pathways, introducing water injection and tracer studies provided valuable insights into how hydrogen might behave under similar conditions. In this study, the systems were initially fully saturated with water, and an injection well was placed to inject a tracer (water) at a constant bottom-hole pressure (BHP) of 20,000 kPa. A well at the opposite end of the model was set to produce water at a constant BHP of 10,000 kPa. Both wells operated simultaneously until the system reached steady-state pressure. At this stage, the water velocity in the grid blocks was evaluated to assess the heterogeneity. Subsequently, a tracer was injected into the system to visualize flow pathways and determine the breakthrough time at the production well. The outcomes of this analysis are presented in the Section 3.

Three sets of simulations were conducted for each permeability map to assess the impact of heterogeneity on the hydrogen storage performance. In the first set of simulations, hydrogen was injected and produced at a constant rate of 3.29 m^3^/day, in which the gas recovery, pressure behavior, and water production were studied. The second stage involved hydrogen injection at a constant BHP of 20,000 kPa, producing hydrogen at a constant rate of 3.29 m^3^/day. Building on the analysis of heterogeneous effects on hydrogen storage, the next operational strategy was developed to determine the optimal injection rate for the first injection. The objective was to avoid exceeding the fracture pressure in the grid blocks, where the injection and production wells are located after 30 days of hydrogen injection into the heterogeneous systems. The cyclic injection and production of hydrogen is summarized in Table 5.

**Table 5:** Operational Strategy of Hydrogen Storage Using the Heterogeneous Model

operational strategy 1	first H_2_ injection (0–30 D)	first production (30–60 D)	second H_2_ injection (60–90 D)	second production (90–120 D)
	3.29 m^3^/day	3.29 m^3^/day	3.29 m^3^/day	3.29 m^3^/day

operational strategy 2	first H_2_ injection (0–30 D)	first production (30–60 D)	second H_2_ injection (60–90 D)	second production (90–120 D)
	BHP of 20,000 kPa	3.29 m^3^/day	BHP of 20,000 kPa	3.29 m^3^/day

operational strategy 3	first H_2_ injection (0–30 D)	first production (30–60 D)	second H_2_ injection (60–90 D)	second production (90–120 D)
	4.15 m^3^/day	4.15 m^3^/day	4.15 m^3^/day	4.15 m^3^/day
	5.55 m^3^/day	5.55 m^3^/day	5.55 m^3^/day	5.55 m^3^/day
	4.25 m^3^/day	4.25 m^3^/day	4.25 m^3^/day	4.25 m^3^/day
	3.29 m^3^/day	3.29 m^3^/day	3.29 m^3^/day	3.29 m^3^/day

The aim of this work is to quantify how saturation-history effects (hysteresis) interact with permeability architecture under controlled conditions. Because publicly available field-scale UHS data sets for porous media remain limited, our Darcy­(reservoir)-scale outputs should be interpreted as scenario-based screening insights rather than history-matched forecasts. This positioning is consistent with the broader UHS literature, which highlights the current scarcity of field benchmarks and the need for progressively coupled experimental–numerical frameworks. To test whether the main conclusions from the 2D areal model survive once a vertical dimension is introduced while keeping the same heterogeneity, we also ran a targeted 3D check using the first strategy on omni-directional permeability realization. The model thickness was increased from 0.5 to 2.5 m with five layers, while replicating the same permeability heterogeneity in each layer to isolate vertical effects without introducing additional vertical geology uncertainty. Five horizontal wells were perforated across all of the layers. Although it is acknowledged that a full 3D description is ultimately required for site-specific design, as buoyancy segregation, vertical layering, and structural trapping can materially affect plume migration and containment, the primary objective of this work is not to deliver a field-calibrated prediction.

## 3 Results and Discussion

The objective of this section is to gain a deeper understanding of gas trapping and its impact on the hydrogen storage efficiency. Two identical homogeneous models were simulated, one with gas relative permeability hysteresis and the other without, employing the relative permeability and capillary pressure data for all hydrogen injection and production cycles (Figures 1 and 2).

### 3.1 Summary

As mentioned in the methodology section, prior to the main simulation, we conducted several sensitivity analyses to ensure that our results are valid across a wide range of conditions.

First, grid sensitivity analysis was performed to confirm the numerical stability. Resolutions coarser than 50 × 400 were insufficient to resolve saturation gradients and mobility-driven front dynamics, leading to distorted water-cut behavior. By contrast, results at 50 × 400 and 100 × 800 were effectively converged for recovery and BHP. We therefore adopted 100 × 800 to track plume advancement with higher spatial fidelity while maintaining practical runtimes; details are provided in the Supporting Information (Figures S1 and S2)

We also compared closed, semiclosed, and pressure-supported (open/constant pressure) boundary conditions. In the closed and semiclosed cases, water has limited ability to redistribute out of the modeled system during injection, so the injection BHP constraint of 20,000 kPa is reached early, and the injection rate is reduced. This yields a smaller injected hydrogen inventory relative to that in the pressure-supported case. During withdrawal, the smaller mobile gas inventory causes the gas rate to collapse earlier, leading to a sharp rise in water cutoff at late times (a ratio effect amplified by the declining gas rate). In the closed and semiclosed cases, water-cut can later decline once local mobile water near the producer is depleted and cannot be replenished effectively, whereas the pressure-supported boundary sustains larger injected volumes and delays the onset of strong water production. The observed jump in water-cut for closed/semiclosed cases is consistent with a reduction in gas rate near the end of the production step rather than a sudden physical change in water encroachment. BHP and water-cut vs recovery factor plots are provided in the Supporting Information (Figures S4 and S5). Since the boundary represents far-field hydraulic communication beyond the near-wellbore domain, we selected an open aquifer as the default boundary condition.

In field-scale compositional simulators such as CMG-GEM, maximum residual gas saturation (*S*_grmax_) is implemented as a rock-function parameter within the relative permeability hysteresis framework, meaning that it can be assigned per rock type without explicitly resolving pore-size distributions; however, each rock type still requires internally consistent drainage and imbibition relative permeability to avoid turning *S*_grmax_ into an unconstrained tuning knob. Our sensitivity analysis based on Carlson’s correlation (*S*_grmax=_ 0.30, 0.35, 0.40) shows that increasing *S*_grmax_ systematically increases the water-cut at a given recovery factor and accelerates the late-time rise in water-cut, consistent with stronger residual trapping reducing the amount of mobile gas available to sustain production as withdrawal progresses. Over the simulated cycles and operating constraints considered here, the primary impact of varying *S*_grmax_ is on water-handling intensity and the timing of water-dominated behavior, whereas the attainable recovery factor remains comparatively less sensitive within this bounded range (Supporting Information, Figure S3)

For the 2D configurations, hydrogen recovery factors in all scenarios have been summarized in Table 6 to make a better comparison between the results. After accounting for hysteresis in the developed model, a considerable amount of hydrogen was trapped in the porous media, resulting in a relatively poor recovery factor during the first production. However, the recovery improved in subsequent cycles, thereby reducing the impact of hysteresis. As can be seen in Table 6, different heterogeneity results in various storage behaviors. Consequently, in large-scale operations, to maximize hydrogen storage efficiency, various key factors should be considered when selecting injection and production flow rates, such as reservoir heterogeneity, petrophysical properties, and water–gas ratio. Additionally, to demonstrate closure and to verify that the cycling protocol does not inadvertently lose hydrogen at the model boundary, we report a cycle-wise hydrogen mass balance based on cumulative injected and produced volumes in the Supporting Information (Table S1).

**Table 6:** Summary of Hydrogen Recovery in Various Scenarios (Standard Condition)

models	1^st^ RF (%)	2^nd^ RF (%)	1^st^ RF % (with 25% water-cut limit)	2^nd^ RF % (with 25% water-cut limit)	1^st^ RF % (after rate optimization)	2^nd^ RF % (after rate optimization)
Homogeneous with no Hysteresis	61.2	82.0				
Homogeneous with Hysteresis	58.4	79.8				
Heterogeneous Omni-Di	59.4	80.4	54.0	79.5	60.3	81.4
Heterogeneous Bi-Di 0°	55.7	77.1	46.6	73.6	56.9	78.5
Heterogeneous Bi-Di 45°	59.1	80.1	54	80.1	60.2	80.2
Heterogeneous Bi-Di 90°	61.9	81.9	61.9	81.9	61.9	81.9

After simulating the 3D models, the BHP response remains consistent across layers and closely matches the 2D case, while gravity segregation is visible through higher water-cut and lower recovery in the deepest layer. When aggregated across layers, the overall 3D performance remains close to the 2D result because the heterogeneity is identical. The results support using 2D to efficiently isolate *X*–*Y* heterogeneity effects, while acknowledging that vertical segregation becomes more influential during any shut-in period, when viscous force weakens, and buoyancy has more time to reorganize the saturation profile, which requires broader 3D structural sensitivity.

On this basis, we retain the 2D model for the main sensitivity matrix: it is the most efficient way to isolate and compare *X*–*Y* heterogeneity architecture without paying a high computational cost for vertical physics that we are not yet parametrizing (e.g., vertical permeability structure, caprock proximity, realistic layering, and rest-time effects). Details and plots are provided in the Supporting Information (Figures S8 and S9).

In this work, we do not optimize a cushion gas strategy; instead, we report gas recovery under the imposed cycling protocol while explicitly modeling hysteresis-driven residual trapping as a physical mechanism that immobilizes gas during imbibition. Gas remaining at the end of a cycle, therefore, comprises both residually trapped gas and gas left in place due to operational constraints (e.g., minimum BHP, water-cut limits, and cycle duration); the latter can serve as an “effective cushion” operationally, but is conceptually distinct from engineered cushion gas. Because UHS cushion gas requirements can be substantial in aquifers and strongly site-dependent, we treat cushion gas design as a separate optimization problem addressed in future studies.

### 3.2 Hysteresis Analysis

In the first injection (initial 30 days), with an injection rate of 6.15 m^3^/day, a total of 29215 m^3^ of hydrogen was injected into the system. Given that the gas and water displacement was based on the primary drainage curve in the first injection in both models (with and without hysteresis effect), a similar volume of hydrogen was injected into the porous media (Figure 6).

**Figure 6:**
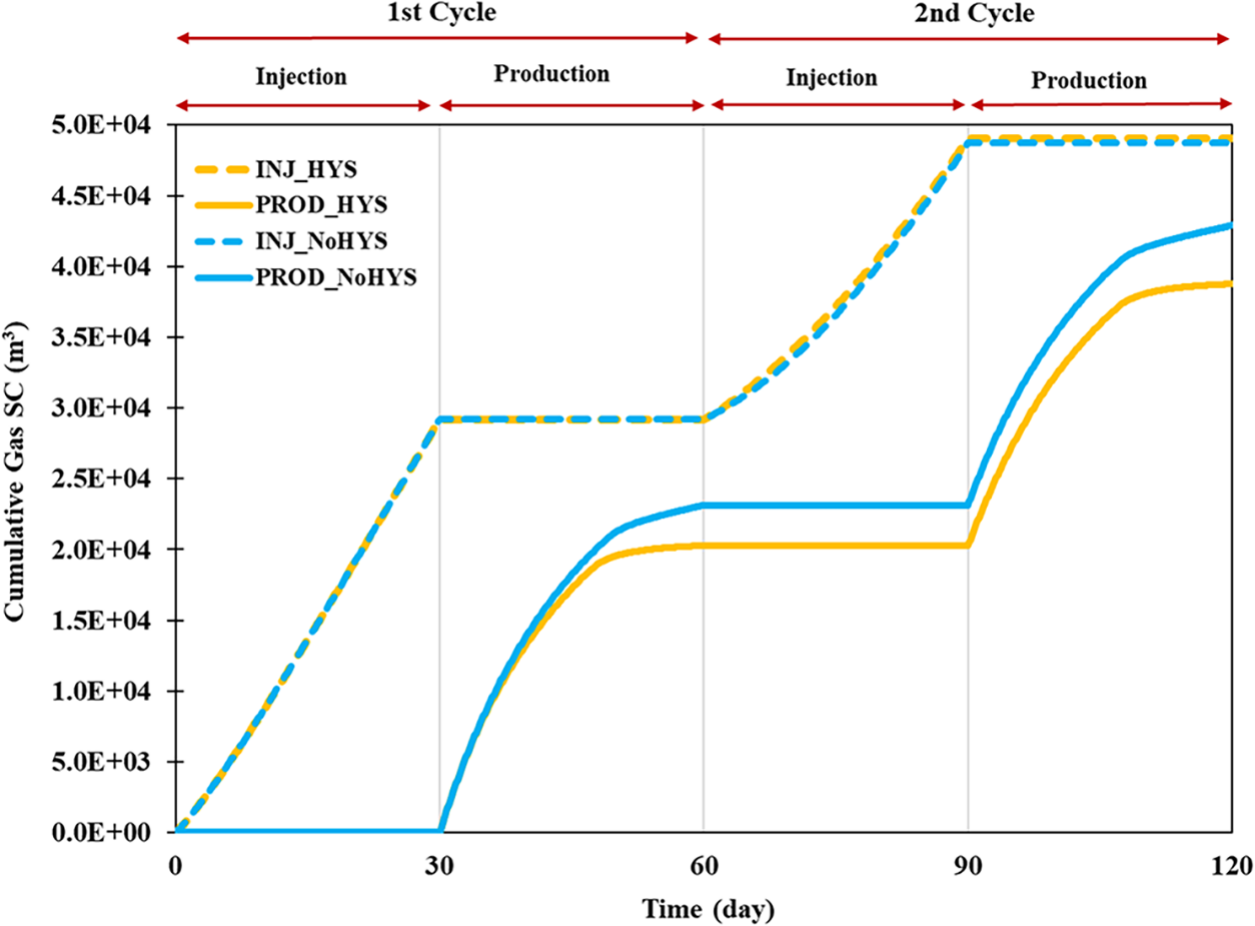
Cumulative produced and injected gas comparison in the homogeneous system.

In the homogeneous system without hysteresis effect, during the subsequent 30 days, 17,895 m^3^ of hydrogen gas was produced. Consequently, the final recovery of primary production reached 61.2%. It is worth noting that cumulative gas production did not reach a steady-state value after 30 days, indicating that hydrogen could not be trapped in the system. If production continued for this case, close to 100% of the injected hydrogen would ultimately be produced. In the second injection (days 60–90), 20,288 m^3^ of hydrogen gas was injected (Figure 6). As before, the final recovery of secondary production at the end of the second production approached 82.0%.

However, given the effect of hysteresis, which leads to gas trapping, different results were anticipated after the first hydrogen injection. In the following 30 days, only 17,058 m^3^ of hydrogen gas was produced, less than in the scenario without the hysteresis effect. Consequently, the final recovery of primary production was 58.4%. Furthermore, after 30 days, cumulative gas production reached a steady-state value, and further production would yield only minimal amounts of gas.

In the second injection, 20,275 m^3^ of hydrogen gas was injected. Due to the residual gas from the previous stage, the gas relative permeability is nonzero and corresponds to the gas saturation. Consequently, hydrogen had a higher mobility than in the first stage, allowing it to reach the spill point more rapidly by achieving greater connectivity (Figure 6). When hydrogen reached the spill point during the second hydrogen injection, a smaller volume was injected into the media. The final recovery of secondary production at the end of the first production reached 79.8%, with slight variations expected if production continues.

Notably, the recovery factor increased in later cycles in both cases. After hysteresis was introduced into the model, a significant amount of hydrogen remained in the system and could not be produced. However, with the second hydrogen injection, mobile gas could move freely through the pores, avoiding entrapment near the wellbore, which had previously contained trapped gas; consequently, a smaller amount of hydrogen was trapped and could not be entirely produced.

In this study, the focus is on relative permeability hysteresis and permeability heterogeneity, excluding clay-specific processes. The current evidence indicates that molecular adsorption of H_2_ on common reservoir clays is small compared with that of other sinks (dissolution and capillary/residual trapping). However, adsorption can be more appreciable in organic-rich or ultratight rocks. The more impactful clay effect is achieved through a change in wettability, which can alter contact angles, alter *k*_r_–P_c_ scanning paths, and increase residual gas saturation during imbibition.^40^ Accordingly, future work should obtain site-specific flow functions for clay-bearing intervals and explicitly account for wettability variability upon scaling to the field.

The water/gas ratio is a key indicator of recovery efficiency and flow dynamics during hydrogen production. The nonhysteresis model’s water–gas ratio remains consistently low across all production cycles, reflecting efficient gas recovery with minimal water production (Figure 7b). This is expected, as the absence of residual gas trapping allows hydrogen to flow freely, maintaining high gas mobility and reducing the relative flow of water. In contrast, the hysteresis model exhibits a significantly higher water–gas ratio, particularly during the first production. This increase is directly attributable to the residual gas saturation (calculated by Land’s correlation), which traps a substantial portion of the injected hydrogen, reducing gas mobility and forcing more water to flow during withdrawal. The higher water–gas ratio in the hysteresis model compared to the nonhysteresis model highlights the operational challenges associated with trapped gas, including higher water-handling costs and the risk of water blockage near production wells. However, the ratio improves slightly in later cycles as residual gas enhances connectivity, allowing hydrogen to mobilize and reducing water production. This cyclic adaptation leads to improved gas movement (mobility) and higher recovery in subsequent cycles, thereby lessening the negative effect of hysteresis over time. The aquifer water influx is another critical metric, reflecting the system’s pressure dynamics and the need for aquifer support to balance pressure changes during production. In the nonhysteresis model, water influx is high to compensate for the pressure by displacing more water into the reservoir, increasing the need for significant aquifer support (Figure 7a). However, the water influx is significantly lower in the hysteresis model, particularly during the first production. The trapped gas reduces the pore space, leading the aquifer to displace less water into the reservoir. This decreased water influx introduces additional operational complexities, such as the need for water management in the storage zone. In later cycles, water influx decreases as residual gas saturation stabilizes, and the aquifer adjusts to the reduced pressure drawdown caused by improved gas connectivity. This trend suggests that while hysteresis initially exacerbates water influx, the system can adapt over time, reducing the need for excessive aquifer support. It is essential to note that in subsequent operations after the initial injection water influx at the aquifer boundary fluctuates due to transient fluid flow. This indicates that pressure does not immediately stabilize throughout the system after hydrogen injection or production begins. Fluid flow direction and pressure changes are more pronounced near the wellbore, while areas near the system’s boundary experience delayed responses. This transient behavior results from the system’s attempt to equilibrate pressure gradients created by injection and production activities.

**Figure 7:**
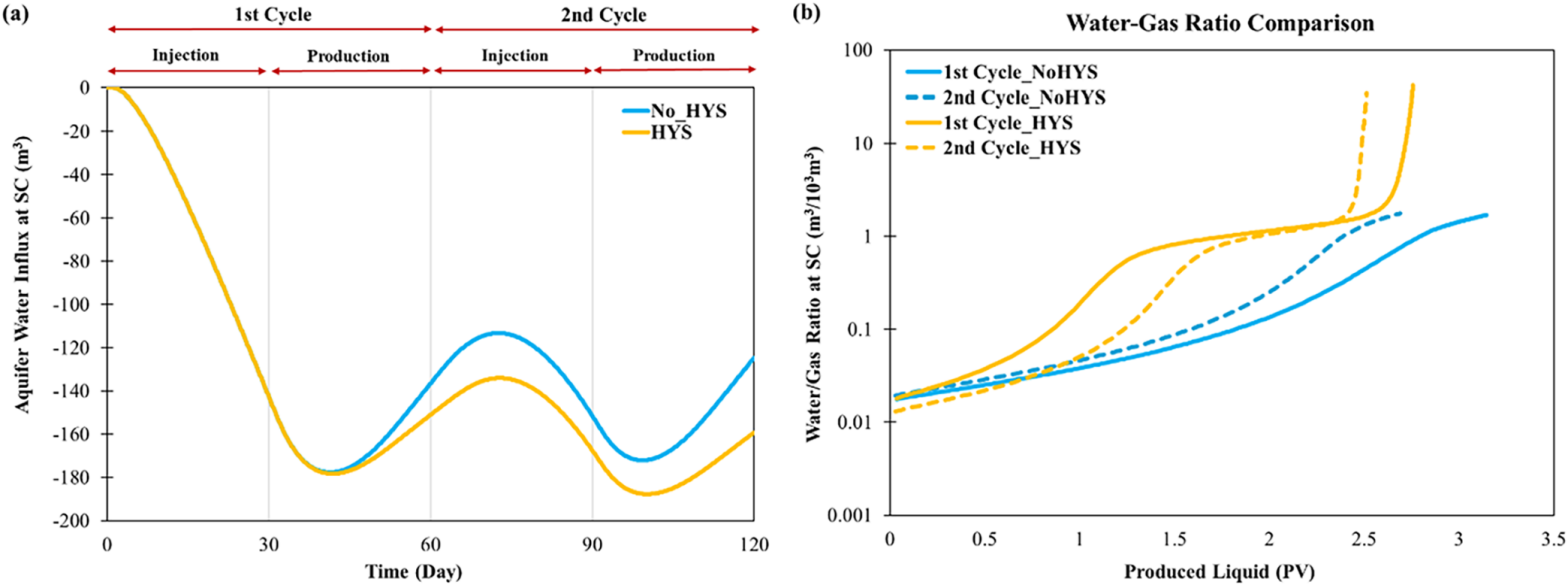
Aquifer water influx (a) and gas/water ratio (b) in the homogeneous system (hysteresis vs nonhysteresis).

To have a better understanding of long-term stabilization, we extended the simulations to four injection and four production cycles. The first production cycle shows the earliest and steepest increase in water-cut at a given recovery factor, while later cycles shift toward lower water-cut for similar recovery levels and achieve higher recovery before water dominance occurs. BHP trajectories become more consistent after the first cycle, with lower peak pressures and stable pressure swings, reflecting saturation-history conditioning and the buildup of an operational gas inventory. The net amount of retained gas, based on the injected and produced volumes, rises quickly at first and then increases more gradually with each cycle, indicating a trend toward a stable cyclic regime under the current constraints. Relevant plots are included in the Supporting Information (Figures S6 and S7).

### 3.3 Heterogeneity Analysis

To evaluate the influence of permeability heterogeneity on fluid flow dynamics, four models (0°, 45°, 90° bidirectional, and omni-directional anisotropy) were analyzed under steady-state pressure conditions. This analysis is used as diagnostics for the displacement regime and connectivity, and not as a direct measurement of trapping. The average water-velocity fields show how each variogram orientation divides displacement into rapid channels versus low-flux areas; we quantify this partitioning through the spread between *v*_min_ and *v*_max_ (and their ratio as a simple indicator of uniformity). The 0° bidirectional model exhibited the shortest breakthrough time and high flow rate, driven by a high degree of fingering through pathways with high permeability (Figure 8b); this configuration risks early breakthroughs and leakage. In contrast, the 90° bidirectional model showed the longest breakthrough time (1,385 days) and lowest flow rate (3.11 m^3^/day), with tortuous flow paths (*v*_min_/*v*_max_ = 0.064) with enhanced sweep efficiency but lower operational feasibility (Figure 8d). The omni-directional and 45° bidirectional models (Figure 8a,c) balanced these extremes, offering moderate breakthrough times and dispersed flow patterns. The homogeneous baseline model, though efficient (flow rate = 6.40 m^3^/day), demonstrated uniform flow, underscoring its impracticality for mimicking natural reservoirs. These results highlight the trade-off between efficiency and security in hydrogen storage, where anisotropic orientation dictates flow behavior. This matters for cyclic UHS because residual trapping is created primarily during imbibition when brine re-enters and disconnects gas, and the severity of this process depends on saturation history and how much of the pore space was bypassed during the preceding displacement. Systems aligned with injection/production axes (e.g., 0°) prioritize rapid recovery but risk containment failure, while perpendicular systems (e.g., 90°) enhance security at the cost of operational efficiency. Future designs must account for anisotropy-driven flow channelization and dispersion to optimize storage integrity and recovery in saline aquifers. The flow characteristics and breakthrough times are summarized in Table 7.

**Figure 8:**
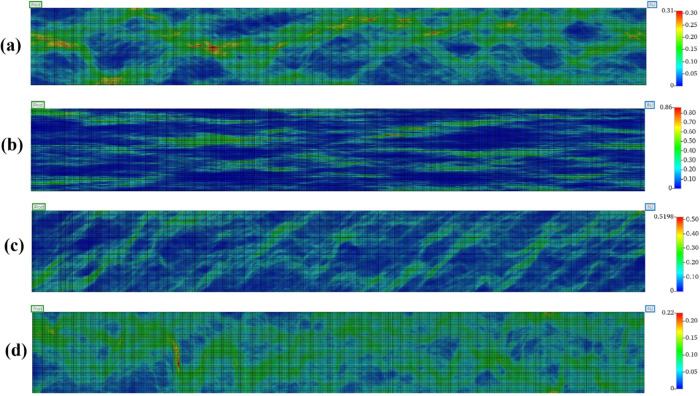
Average water velocity map. (a) Omni-directional continuity, (b) bidirectional continuity with an angle of 0°, (c) bidirectional continuity with an angle of 45°, and (d) bidirectional continuity with an angle of 90°.

**Table 7:** Flow Characteristics and Breakthrough Times for Heterogeneous and Homogeneous Models When Water Was Injected into the System under Steady-State Conditions

model	breakthrough time (day)	flow rate (m^3^/day)	*v*_ave_ in grid blocks (m/day)	*v*_max_ (m/day)	*v*_min_ (m/day)	*v*_max_/*v*_min_
BiDi0	475	5.740	0.114	0.863	0.003	0.004
BiDi45	925	4.170	0.085	0.520	0.009	0.017
BiDi90	1385	3.110	0.064	0.225	0.014	0.064
Omni-Di	825	3.970	0.081	0.310	0.010	0.031
Homogen	875	6.400	0.126	0.126	0.126	1.000

Analyzing heterogeneous systems provides critical insights into how permeability variation influences hydrogen storage efficiency, particularly in terms of recovery factors, pressure dynamics, and water management. The flow-aligned system (0° bidirectional) exhibits the lowest recovery factors (55.7% in Cycle 1 and 77.1% in Cycle 2) and stable bottom-hole pressure, indicating inefficient hydrogen flow. This is attributed to the alignment of the highest tortuous flow paths with the flow direction, facilitating rapid hydrogen migration and increased trapping. In contrast, systems with perpendicular heterogeneity (90° bidirectional) exhibit the highest recovery (61.9% in Cycle 1 and 81.9% in Cycle 2) due to restricted flow paths. However, this model exhibits higher pressure variations during hydrogen injection and production cycles than other anisotropic configurations (Figure 9). This heightened pressure fluctuation is primarily due to the restricted flow of hydrogen through the pathways, leading to significant pressure buildup during injection and sharp declines during production. While this model achieves higher recovery factors (Figure 10), the pronounced pressure changes pose a risk to reservoir integrity, particularly in scenarios involving high cycling rates. When the bottom-hole pressure exceeds the designed upper-pressure limit or the minimum horizontal stress, tensile damage can occur, activating existing fractures or generating new ones.^41^ Tensile fractures, which are more severe than shear damage in shallow reservoirs,^42^ can alter permeability and permeability anisotropy,^43^ potentially accelerating hydrogen migration and compromising storage security. Therefore, accurately evaluating the tensile strength and in situ stress is critical to mitigate these risks. While much of the existing knowledge on cyclic stress effects comes from natural gas storage, the unique properties of hydrogen, such as its lower viscosity and higher diffusivity, may aggravate these challenges. Therefore, further research is needed to understand how hydrogen-specific stress alterations affect reservoir and caprock behavior, fracture propagation, and long-term storage integrity. The behavior of 45° bidirectional and omni-directional models is similar, striking a balance that achieves a high recovery in later cycles (80% and 80.4%, respectively, in the first and second cycles), while experiencing moderate pressure fluctuations.

**Figure 9:**
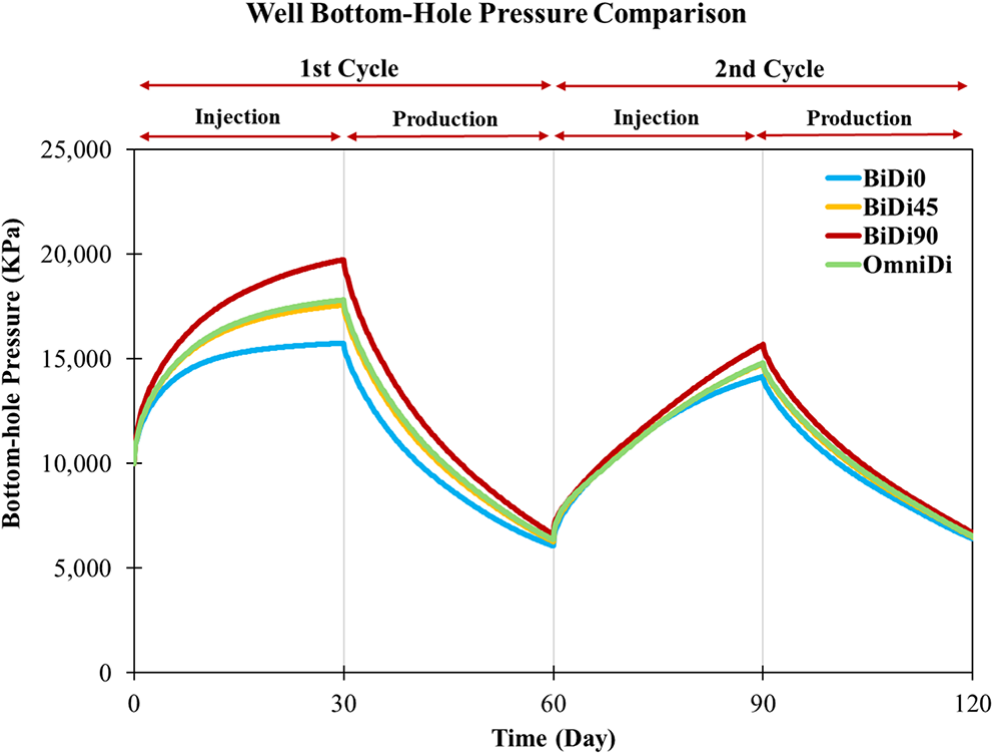
Well bottom-hole pressure comparison in the heterogeneous systems under a constant gas injection rate of 3.29 m^3^/day.

**Figure 10:**
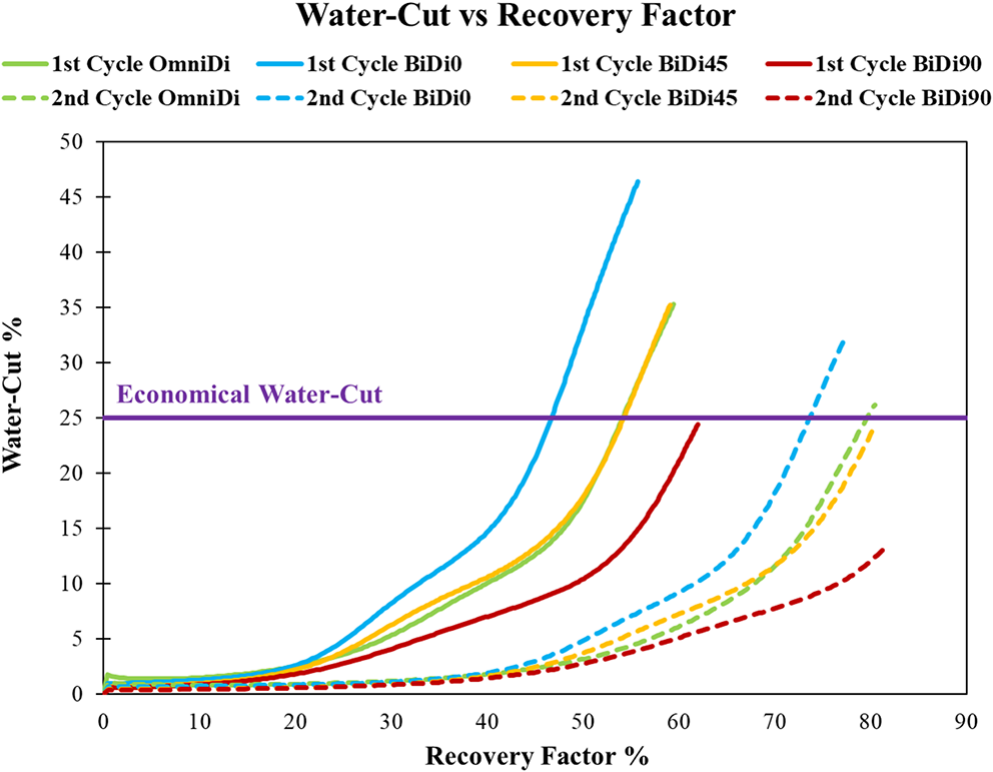
Water-cut vs recovery factor comparison in the heterogeneous systems under a constant gas injection rate of 3.29 m^3^/day.

Figure 10 illustrates the water cutoff (water ratio to total produced fluids) during withdrawal across heterogeneous models, revealing a critical trade-off between hydrogen recovery efficiency and water management. The 0° bidirectional model, characterized by rapid flow along high-permeability pathways, exhibits the highest water-cut in both cycles. This aligns with previous findings that flow-aligned systems exacerbate water production due to preferential flow, necessitating interventions such as artificial lift or water shutoff when water cuts exceed 70%. In contrast, the 90° bidirectional model demonstrates the lowest water-cut, consistent with its slower flow dynamics and lower water production rates.

The 45° bidirectional and omni-directional models strike a balance, maintaining moderate water cuts (∼25–35%) while achieving reasonable gas recovery. Notably, the decreased water-cut in later cycles suggests a reduction in the water relative permeability, a phenomenon attributed to residual gas saturation altering flow pathways. These trends underscore the necessity for tailored operational strategies, such as controlled withdrawal rates or cyclic pressure management, to optimize recovery while minimizing water-handling costs. A 25% water-cut threshold was adopted based on typical operational limits observed in conventional gas reservoirs, where higher water production can significantly increase handling and disposal costs. As noted by Guo et al., gas wells often become uneconomical when water-cut exceeds approximately 20–25%, primarily due to increased separation and treatment requirements.^44^ It should be noted that there is no widely accepted case history in the hydrogen storage field that defines a standard economic water-cut limit. Hydrogen-specific surface-handling costs are uncertain, and integrity/material considerations (including risk management) can shift the trade-off between recoverable gas and water processing. Therefore, the 25% water-cut value is used as an illustrative operational screening constraint and not a hydrogen-specific economic optimum. Hydrogen’s value may justify tolerating higher water-cut than conventional natural gas, but surface economics are outside the scope of this study and require dedicated techno-economic work. Under these constraints, hydrogen recovery factors were notably lower than in unconstrained scenarios, highlighting the importance of water management in optimizing storage performance. For instance, in the 0° bidirectional model, adhering to an economical water-cut limits recovery factors to 46% and 73% in the first and second production cycles, respectively (Figure 10).

The anisotropic ratio quantifies directional permeability variability and is a critical parameter influencing flow behavior. To mitigate water management challenges, a thorough evaluation of this ratio is essential prior to hydrogen storage operations. Optimal injection and production rates must be designed by integrating cyclic operational phases, porous media heterogeneity, and fluid relative permeability. This approach ensures efficient storage operations with controlled water production, balancing recovery efficiency with long-term reservoir integrity.

In addition to the fixed-rate baseline (3.29 m^3^/day) used to isolate heterogeneity effects at constant throughput, we tested a pressure-controlled injection scenario in which the injector bottom-hole pressure was held at 20,000 kPa (a proxy for integrity window). Under pressure control, injected gas volume becomes an emergent outcome of model injectivity, so higher-connectivity architectures admit more hydrogen than the volume-matched fixed-rate baseline. Under these constraints, hydrogen recovery factors were notably lower than those in the previous scenario (around 50% in the first cycle and 40% in the second cycle). Consequently, withdrawal must be reoptimized to the updated gas inventory and saturation history if the objective is to maximize recovery while maintaining the 25% water-cut operational threshold; the next fixed-rate withdrawal settings are no longer optimum once injection is BHP-limited. This is reflected in the distinct water-cut versus recovery behavior across the four heterogeneity orientations in the pressure-controlled case (Figure 11), where differences in gas migration during injection translate into different water-encroachment patterns during withdrawal.

**Figure 11:**
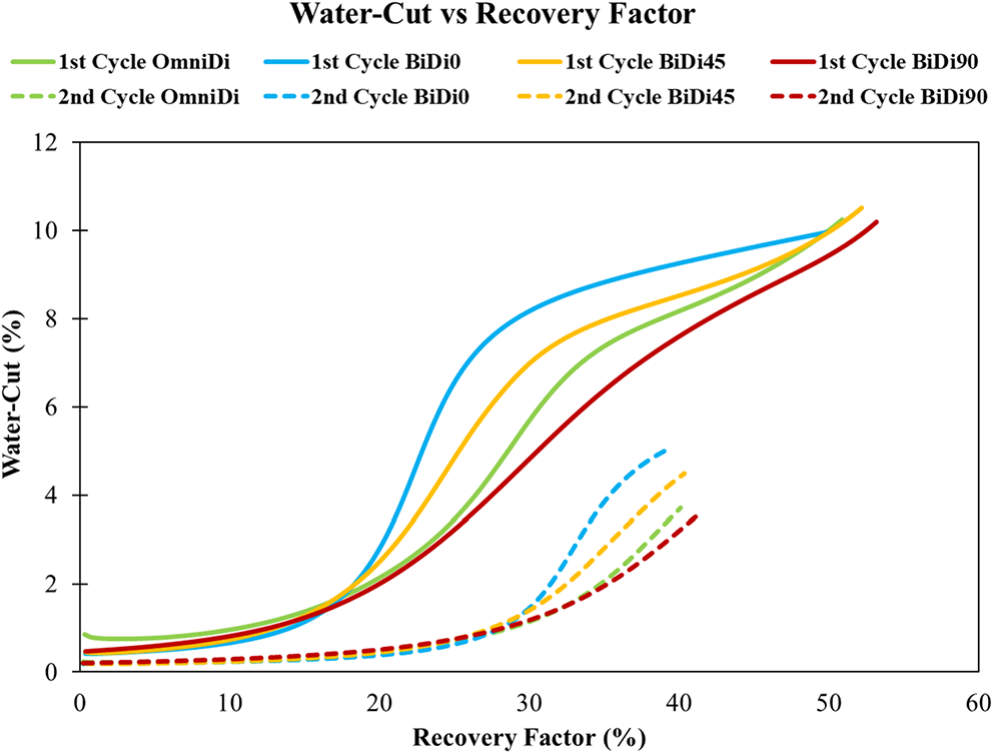
Water-cut vs recovery factor comparison in the heterogeneous systems under a constant injector bottom-hole pressure at 20,000 kPa.

It is also vital to note that pressure-controlled gas injection in underground gas storage might be operationally ideal. In practice, injection rates can be constrained by upstream hydrogen supply from nearby generation facilities (and by allowable wellbore and formation pressure limits), while withdrawal rates are dictated by demand, export capacity, and deliverability constraints. A pressure-controlled injection phase is therefore a reasonable proxy for operating within an integrity window, just as a demand-driven withdrawal schedule is a reasonable proxy for market-led dispatch; the resulting rates are emergent outcomes of supply–demand and subsurface deliverability rather than arbitrary modeling choices.

### 3.4 Flow Rate Optimization

The next step was to optimize the flow rate for each model. The consistent pressure behavior across all models after rate optimization reflects a balance between tailored injection rates and permeability anisotropy (Figure 12). This calibration ensures that pressure remains within the 20,000 kPa fracturing threshold and 5000 kPa, homogenizing pressure trajectories despite differing permeability structures. The convergence of pressure profiles indicates that rate adjustments compensate for inherent flow advantages or restrictions, achieving equilibrium where no single model overstresses the reservoir.

**Figure 12:**
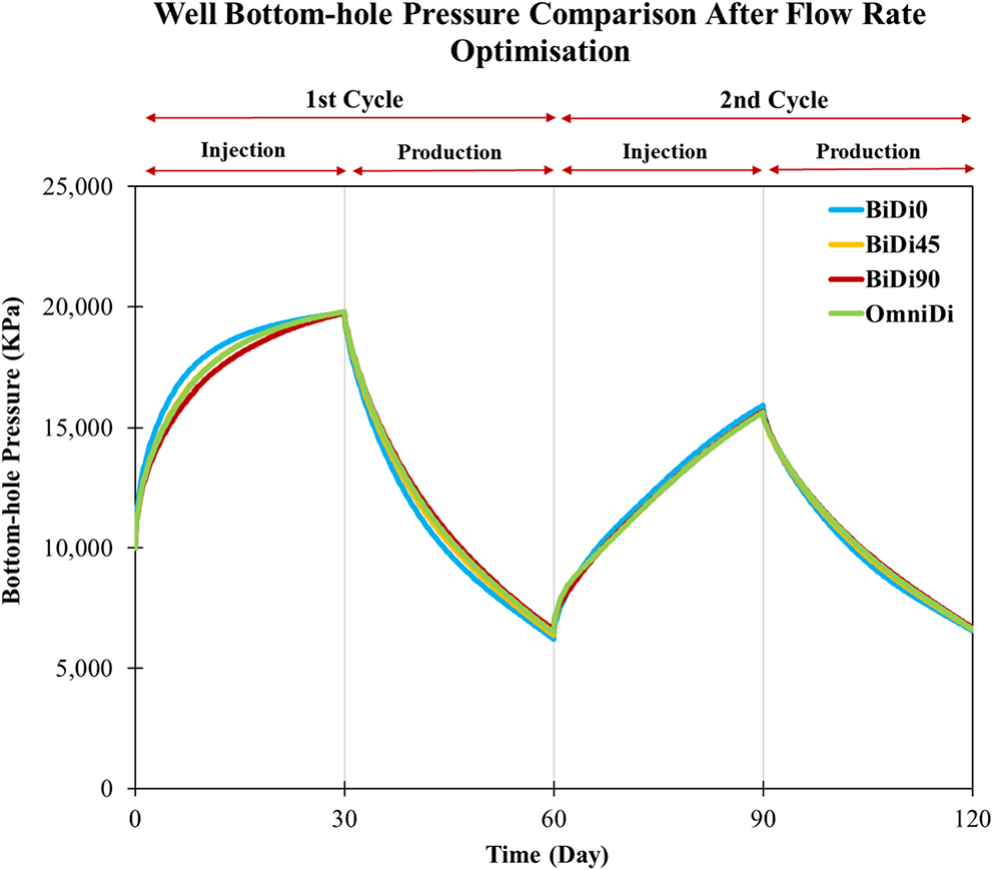
Well bottom-hole pressure comparison in the heterogeneous after the flow rate optimization.

As mentioned before, water production is a vital parameter to consider when developing predictive models.^45^ After optimizing the flow rate, the water-cut does not reach the economic threshold in all models. The nonconstant water-cut slope during production arises from the complex interplay among permeability heterogeneity, gas-migration dynamics, and hysteresis effects. During hydrogen injection, gas preferentially invades high-permeable grid blocks near the well, forming localized “gas banks” that dominate early production. Initially, these zones yield high gas recovery with minimal water-cut. However, as these high-permeability regions deplete, production shifts to lower-permeability areas, where gas mobility is reduced. Water from adjacent brine-saturated zones encroaches into these pathways, gradually increasing the water-cut (Figure 13). This transition from high-to-low permeability drainage creates a nonlinear slope, as water influx accelerates once gas production becomes constrained by less permeable regions and gas saturation decreases. For example, in flow-aligned systems like Bidirectional 0°, rapid gas withdrawal from preferential pathways leaves behind isolated gas clusters that later reconnect, potentially leading to moderate increases in water-cut. Conversely, in restricted systems like Bidirectional 90°, since water was displaced uniformly during injection, there is little change in the water-cut slope, and delayed gas mobilization allows sudden connectivity of residual gas, sharply reducing water-cut in later cycles (Figure 13d).

**Figure 13:**
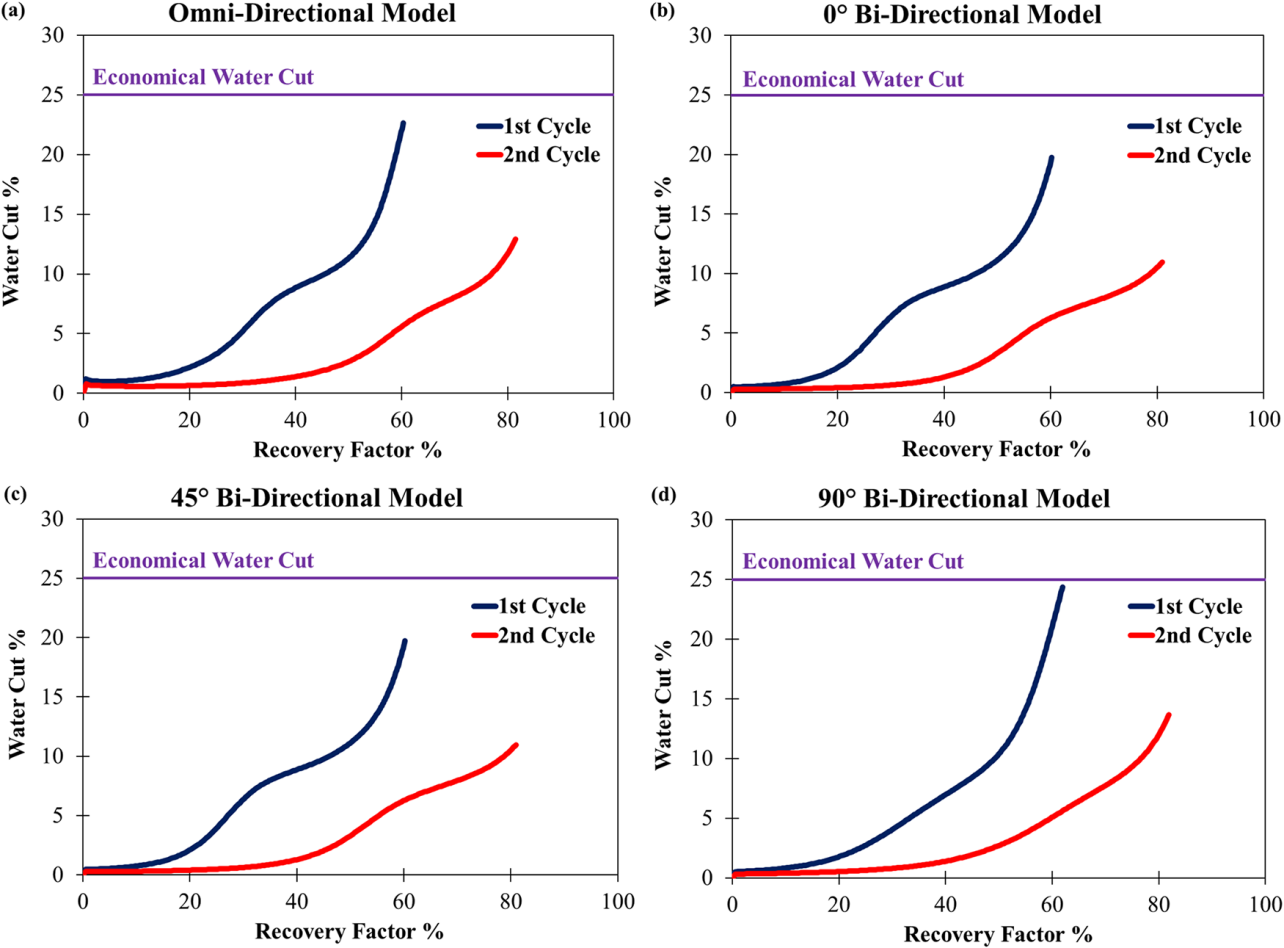
Water-cut vs recovery factor comparison in the heterogeneous systems under optimized gas injection rate. (a) Omni-directional continuity, (b) bidirectional continuity with an angle of 0°, (c) bidirectional continuity with an angle of 45°, and (d) bidirectional continuity with an angle of 90°.

Hysteresis further modulates water-cut dynamics by reshaping pore-scale flow regimes over successive cycles. As the operation progresses, trapped gas reorganizes into connected pathways, enhancing gas mobility and displacing water more efficiently. This hysteresis-driven evolution explains why water-cut slopes vary between the heterogeneous systems; a sharper increase in restricted systems such as bidirectional 90° (Figure 13d) versus gradual improvements inflow-aligned systems such as bidirectional 0° (Figure 13b). Optimized rates ensure recovery efficiency improves without destabilizing pressure and keep water production under an economic threshold. Together, these mechanisms highlight the necessity of cycle-aware operational strategies to manage water production and maximize storage performance in heterogeneous reservoirs.

Beyond engineered storage, the same multiphase transport arises where hydrogen accumulates naturally or is generated in situ. Observations from emerging natural-hydrogen reservoirs show association with water-bearing formations beneath effective seals, with persistence controlled by the balance between generation and losses (diffusion, microbial consumption); deliverability is therefore governed by H_2_–brine flow through heterogeneous media.^46^

In proposed “hydrogen-farming” schemes, reactions within aquifers or reservoirs create H_2_ that must nucleate/exsolve into brine-filled pores, and migrate to wells, so rate/pressure management, water-cut constraints, and connectivity in anisotropic media remain primary operational levers.^46^ Extending the present framework to these applications is straightforward conceptually by adding a spatially distributed source term and coupling reactive transport (abiotic and microbial). Since gas saturation arises by exsolution rather than injection, exsolution-aware *k*_r_–P_c_ and wettability characterization should be used to select appropriate scanning paths and quantify recoverability.

## 4 Conclusions

The efficiency of underground hydrogen storage is critically controlled by the interplay between geological heterogeneity and fluid dynamics, specifically relative permeability hysteresis. Our numerical modeling demonstrates that these factors are not secondary considerations but primary drivers of hydrogen loss and recovery. The key findings and their implications are as follows:

- A significant residual gas saturation was observed, confirming that a substantial portion of hydrogen could become trapped in the rock pores.
- Trapped hydrogen from initial cycles could get connected to the main gas stream in the subsequent injections and get mobilized. Consequently, in later cycles, hydrogen will reach the spill point faster.
- Permeability heterogeneity and its orientation directly govern storage performance. Formation with flow-aligned anisotropy resulted in the poorest hydrogen recovery and highest water production. Although the system with anisotropy perpendicular to the flow showed slightly improved recovery, the sharp pressure variations could threaten reservoir integrity.
- Operational strategies must be tailored to specific geology. By adjusting injection and production rates in heterogeneous models, we successfully maintained reservoir pressures below fracturing thresholds and stabilized water influx, demonstrating that site-specific management is essential for balancing efficiency and safety.
- Managing water production is a critical economic and operational challenge. In our simulations, adhering to a practical water-cut limit of 25% reduced total hydrogen recovery by 5–10%, underscoring the trade-off between resource recovery and water-handling costs.

These insights should be regarded not as a direct prediction of field performance but as a foundational step toward derisking UHS projects. The methodology presented here offers an improved tool for initial screening and characterization of potential storage sites, highlighting the need to move beyond static assumptions.

## 5 Limitations and Future Work

This study focuses on isothermal multiphase flow controlled by relative permeability/capillary pressure hysteresis and permeability heterogeneity architecture during short injection–production cycles. We therefore neglected molecular diffusion and geochemical/biogeochemical reactions. These processes may become increasingly relevant over longer storage durations. They could reduce the effective working gas by transferring a fraction of H_2_ into the aqueous phase (via solubility) and enabling microbially mediated consumption or mineral reactions that alter gas composition and injectivity. Recent reviews highlight diffusion, dissolution, and geochemical/microbial effects as key knowledge gaps and potential loss mechanisms for porous-media UHS, motivating coupled reactive-transport/biogeochemical extensions of the present workflow.^4,6,11^ Also, thermal effects (Joule–Thomson and heat exchange) can become important near the wellbore at high rates/large drawdown, and recent UHS studies quantify wellbore-driven temperature offsets at typical storage depths that can serve as boundary conditions for nonisothermal reservoir simulations.^47^ Importantly, by neglecting these processes, our recovery factors should be interpreted as upper-bound, flow-controlled estimates for the tested cycle durations; incorporating these mechanisms would be expected to further reduce recoverable H_2_ depending on the salinity, temperature, pressure, and residence time. Additionally, future research should explore the effects of hydrogen-specific stress on fracture propagation and caprock integrity, leveraging advanced characterization techniques.

## Supplementary Material



## Notes

Additional results and discussion regarding grid sensitivity analysis, maximum residual gas saturation sensitivity analysis, aquifer’s boundary conditions, pressure optimization in later cycles and comparison study of 3D models with 2D models; hydrogen volumetric balance in the standard condition table for all scenarios (cumulative gas injection and cumulative gas production) (PDF)
