# Supplementary material for: Coupled Hysteresis and Variogram-Controlled Heterogeneity Effects on Underground Hydrogen Storage in Saline Aquifers
Source: Energy Fuels. 2026 Apr 26;40(18):10223–39. doi: 10.1021/acs.energyfuels.6c00987 (PMC13158903; doi:10.1021/acs.energyfuels.6c00987)
Supplement: Supplementary file 1 [file ef6c00987_si_001.pdf]

Supporting Information for:

Coupled Hysteresis and Variogram-Controlled Heterogeneity Effects on Underground Hydrogen Storage in Saline Aquifers

Abdolali Mosallanezhad[1], Amir Jahanbakhsh[1,2*], Azim Kalantariasl[3] and M. Mercedes Maroto-Valer[1,2]*

[1]Research Centre for Carbon Solutions (RCCS), School of Engineering and Physical Sciences, Heriot-Watt University, Edinburgh, EH14 4AS, UK

[2]Industrial Decarbonisation Research and Innovation Centre (IDRIC), Heriot-Watt University, Edinburgh, EH14 4AS, UK

[3]Department of Petroleum Engineering, School of Chemical and Petroleum Engineering, Shiraz University, Shiraz, Iran

*Corresponding authors: am3053@hw.ac.uk, a.jahanbakhsh@hw.ac.uk

Table S1. Full hydrogen volumetric balance in the standard condition table for all scenarios; In the reported runs, cycle-wise in-situ gas is the difference between cumulative injected and produced volumes, consistent with the monitoring-limited protocol that terminates injection upon H_2 detection at the centre observation well.

Scenario	Cycle	Injected H_2 (m³, std)	Produced H_2 (m³, std)	In-situ H_2 (m³, std)	In-situ fraction (%)	Net boundary loss (m³, std)
Homogeneous (no hysteresis)	1	29215.4	17895.1	11320.2	38.7	0
Homogeneous (no hysteresis)	2	20288.6	16637.5	3651.1	18	0
Homogeneous (with hysteresis)	1	29215.4	17058.6	12156.8	41.6	0
Homogeneous (with hysteresis)	2	20275.8	16172.6	4103.1	20.2	0
Heterogeneous OmniDi (Constant Rate)	1	14081.3	8373.5	5707.8	40.5	0
Heterogeneous OmniDi (Constant Rate)	2	10332.6	8307.6	2025.1	19.6	0
Heterogeneous OmniDi (Constant BHP)	1	25326.6	12924.3	12402.3	49	0
Heterogeneous OmniDi (Constant BHP)	2	38690.7	15556.1	23134.6	59.8	0
Heterogeneous OmniDi (Optimised Rate)	1	19293.8	11638.2	7655.6	39.7	0
Heterogeneous OmniDi (Optimised Rate)	2	13505.6	11000.6	2505	18.5	0

Scenario	Cycle	Injected H_2 (m³, std)	Produced H_2 (m³, std)	In-situ H_2 (m³, std)	In-situ fraction (%)	Net boundary loss (m³, std)
Heterogeneous BiDi0 (Constant Rate)	1	12996.3	7239.3	5757	44.3	0
Heterogeneous BiDi0 (Constant Rate)	2	10200.1	7865.9	2334.2	22.9	0
Heterogeneous BiDi0 (Constant BHP)	1	32264.3	16096.7	16167.6	50.2	0
Heterogeneous BiDi0 (Constant BHP)	2	50117.5	19776.1	30341.4	60.5	0
Heterogeneous BiDi0 (Optimised Rate)	1	26261.5	14955.1	11306.4	43	0
Heterogeneous BiDi0 (Optimised Rate)	2	18377.4	14419	3958.3	21.5	0
Heterogeneous BiDi45 (Constant Rate)	1	13968.7	8250.8	5717.9	40.9	0
Heterogeneous BiDi45 (Constant Rate)	2	10332.7	8276.8	2055.9	19.9	0
Heterogeneous BiDi45 (Constant BHP)	1	24994.3	13043	11951.3	47.8	0
Heterogeneous BiDi45 (Constant BHP)	2	39113.2	15777.6	23335.6	59.7	0
Heterogeneous BiDi45 (Optimised Rate)	1	19769.6	11909	7860.5	39.8	0
Heterogeneous BiDi45 (Optimised Rate)	2	13831.6	11194.1	2637.5	19	0
Heterogeneous BiDi90 (Constant Rate)	1	15088.5	9346.9	5741.6	38	0
Heterogeneous BiDi90 (Constant Rate)	2	10697.7	8763.1	1934.6	18.1	0

Scenario	Cycle	Injected H_2 (m^3, std)	Produced H_2 (m^3, std)	In-situ H_2 (m^3, std)	In-situ fraction (%)	Net boundary loss (m^3, std)
Heterogeneous BiDi90 (Constant BHP)	1	19399.1	10319.5	9079.6	46.8	0
Heterogeneous BiDi90 (Constant BHP)	2	29983	12371.8	17611.2	58.7	0
Heterogeneous BiDi90 (Optimised Rate)	1	15088.5	9346.9	5741.6	38.1	0
Heterogeneous BiDi90 (Optimised Rate)	2	10697.7	8763.1	1934.6	18.1	0

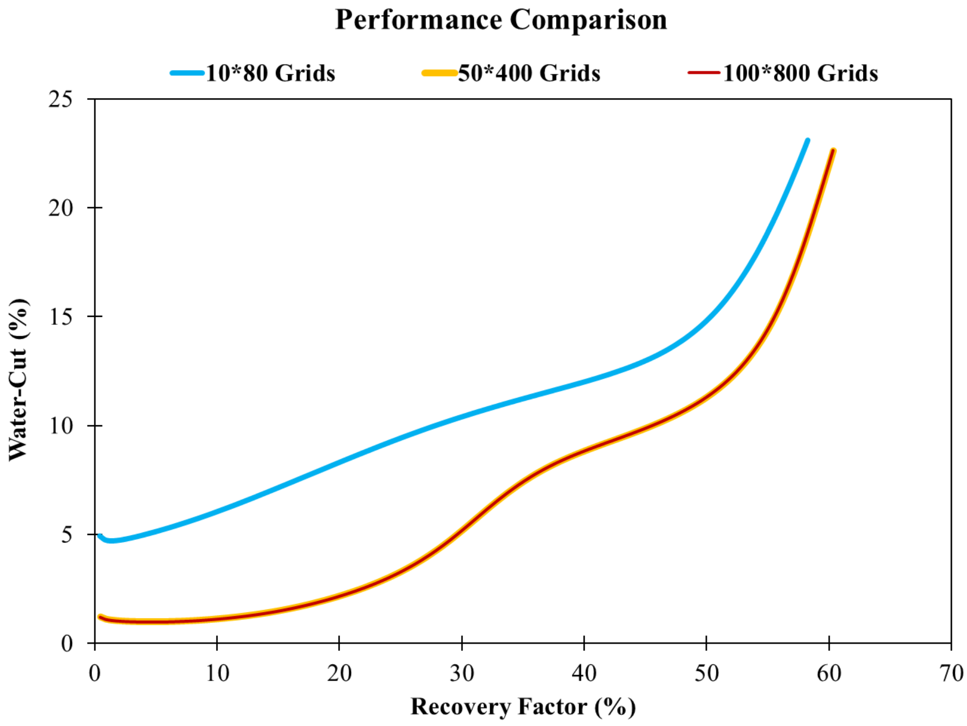

Figure S1. Water-cut versus recovery factor (grid sensitivity). Comparing water-cut evolution against recovery factors for three grid resolutions (10×80, 50×400, 100×800) with identical properties, boundary conditions, and controls. The coarsest grid (10×80) predicts a higher water-

cut due to under-resolved saturation gradients and numerical dispersion. Results at 50×400 and 100×800 nearly overlap, showing convergence of saturation metrics.

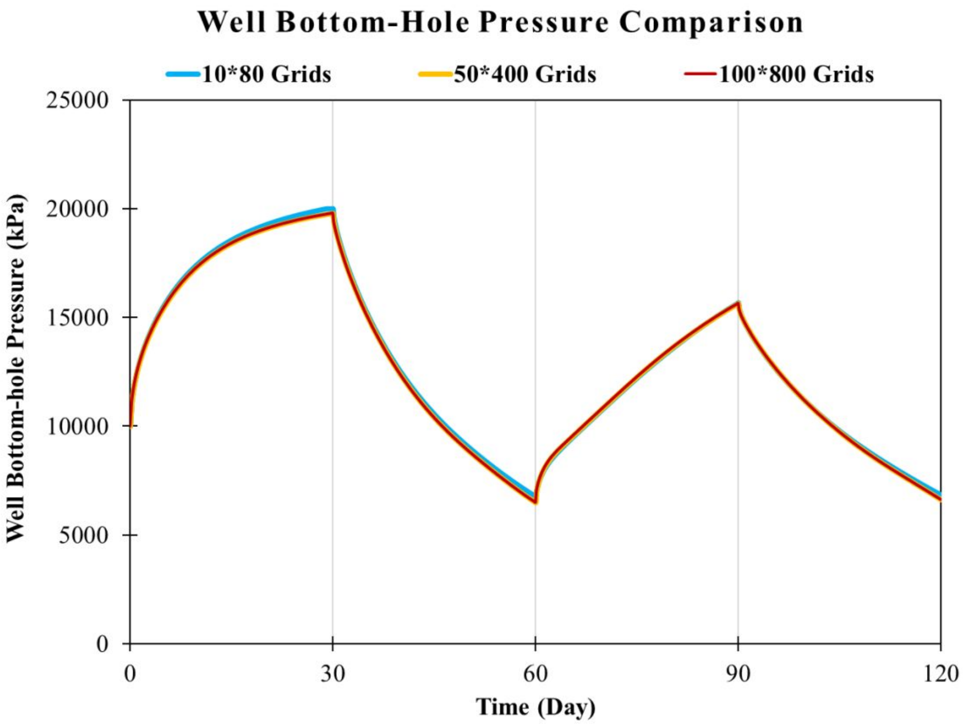

Figure S2. Injector/producer BHP histories for three grid resolutions (10×80, 50×400, 100×800) under the same schedule and pressure constraints show that BHP curves are grid independent. Pressure evolution mainly depends on well controls and boundary support, whereas main grid sensitivity appears in saturation-dependent outputs, such as water cut, rather than in pressure response.

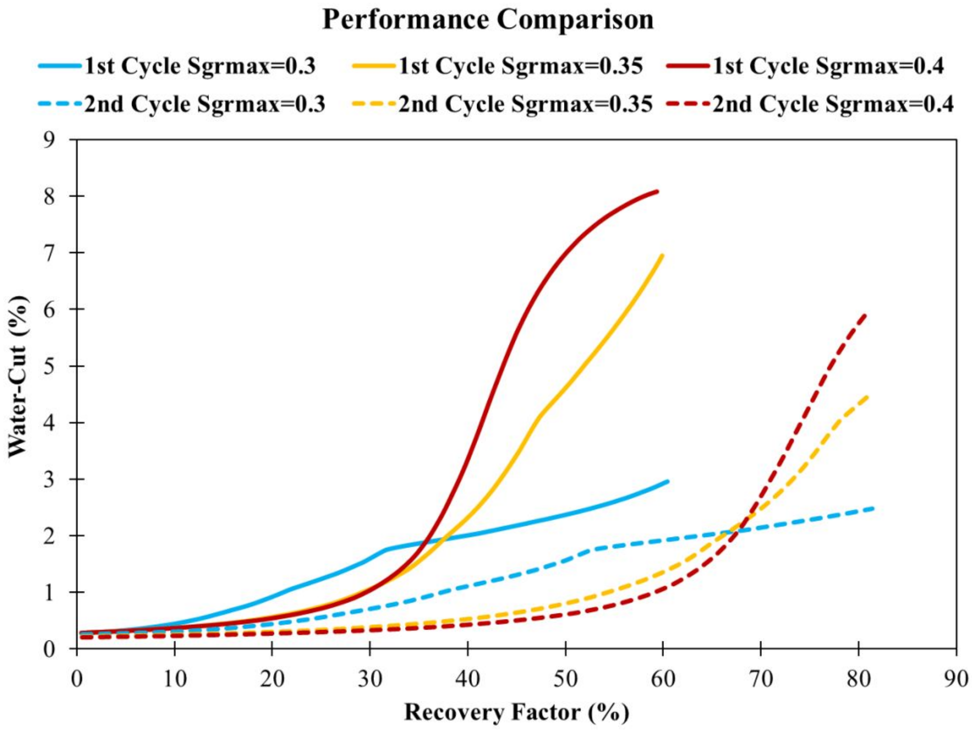

Figure S3. Water-cut vs recovery factor for two production cycles was calculated using three maximum residual gas saturation values (S_{grmax} = 0.30, 0.35, 0.40). Increasing S_{grmax} raises the curves and steepens the late-time water-cut rise, indicating more trapping and less mobile gas connectivity, accelerating the transition to water-dominated production. The second cycle shows similar dependence but reflects prior saturation history (based on Carlson's relative permeability hysteresis).

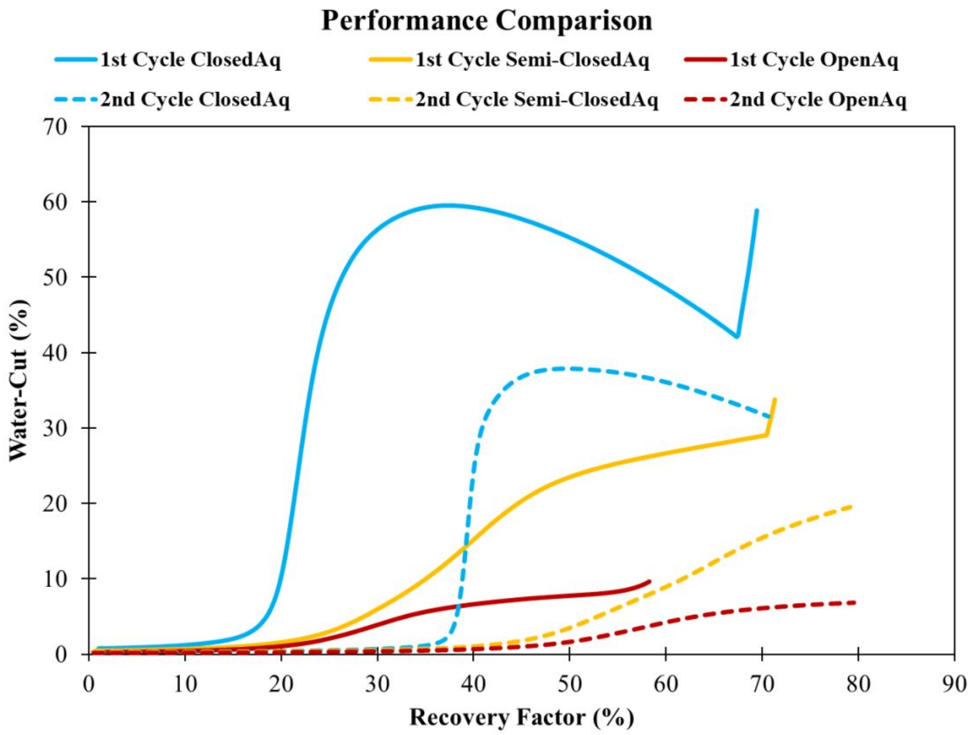

Figure S4. Water-cut vs recovery factor for boundary-condition sensitivity (open, semi-closed, closed). The trajectories show that closed and semi-closed boundaries reach the injection BHP constraint earlier, reducing injected gas and causing earlier gas-rate collapse during withdrawal, with a sharper late-water-cut increase at a given recovery. In closed/semi-closed cases, water-cut may decline after local water near the producer is depleted and not replenished, while open boundaries sustain pressure support and delay water-dominated production.

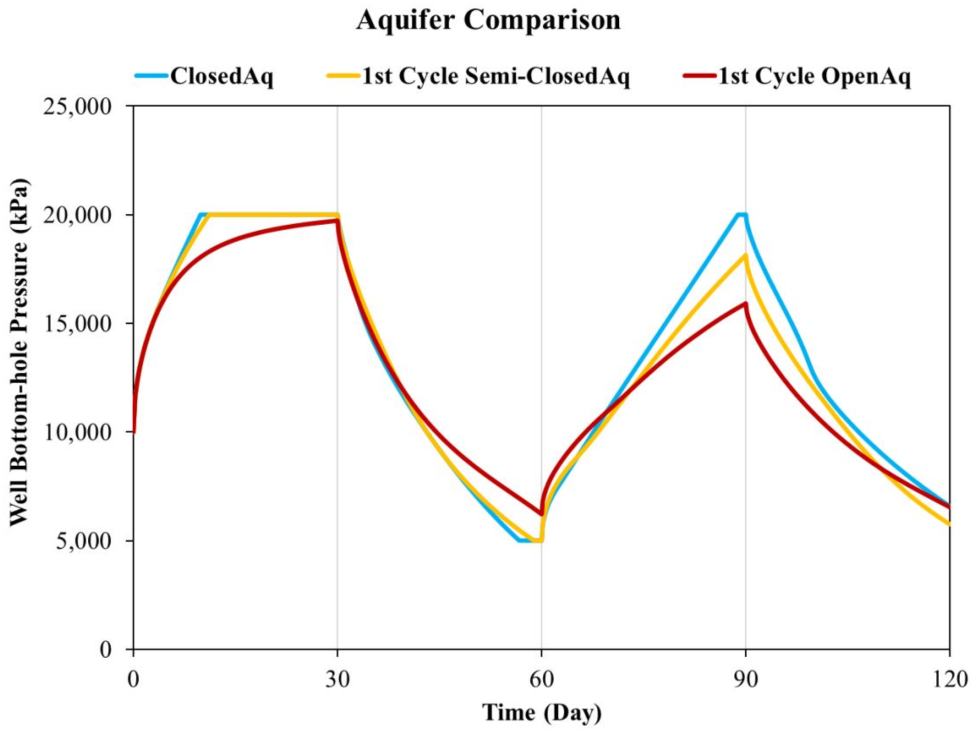

Figure S5. Well bottom-hole pressure comparison for boundary condition sensitivity (open vs semi-closed vs closed). Closed and semi-closed cases approach the injection pressure limit earlier and more persistently, forcing an automatic reduction in injection rate and lowering injected gas volumes relative to the pressure-supported case. During production, the reduced injected inventory and limited pressure support lead to stronger late-time rate constraints and contribute to the end-of-cycle water-cut escalation observed in Figure S4.

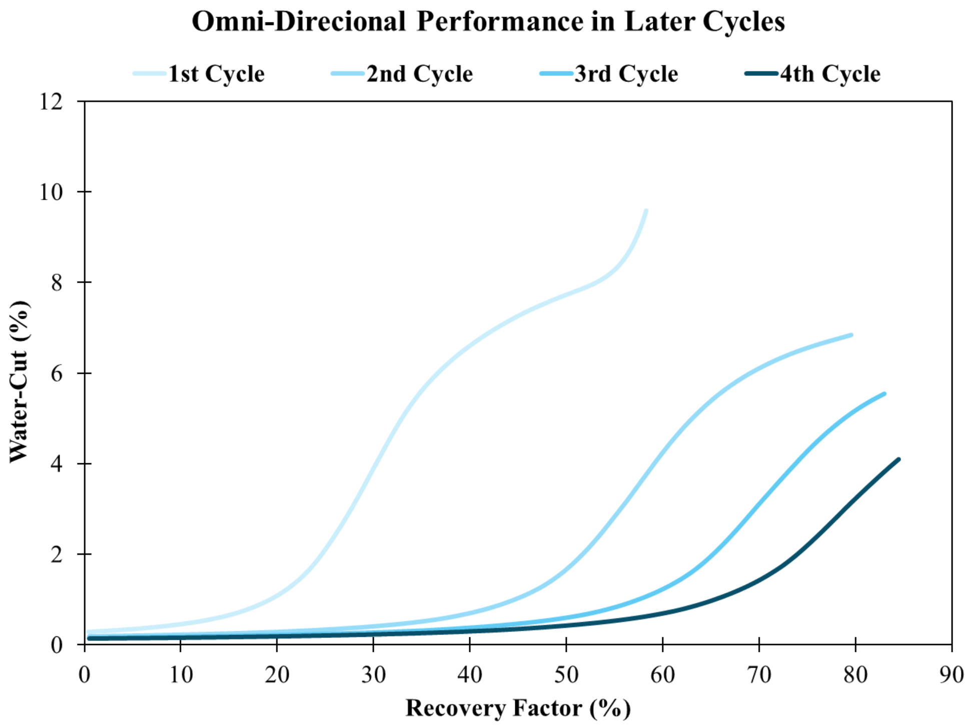

Figure S6. Omni-directional model: water-cut vs recovery factor across cycles (cycle stabilisation). The first cycle shows the earliest and steepest rise in water-cut, while later cycles shift toward lower water-cut at a given recovery factor, indicating progressive saturation-history conditioning and increasingly repeatable cyclic behaviour.

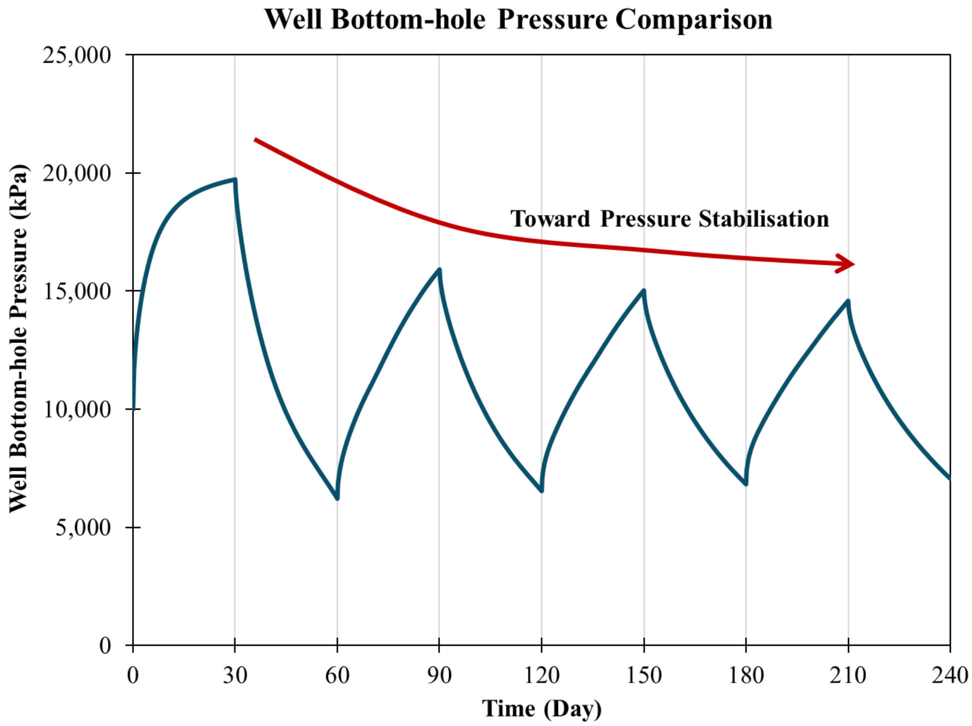

Figure S7. Omni-directional model: well bottom-hole pressure histories across cycles (cycle stabilisation). After the initial cycle, the pressure response becomes more repeatable, with reduced cycle-to-cycle variability, consistent with the development of an internal gas inventory and stabilisation of the flow pathways that govern deliverability under cycling.

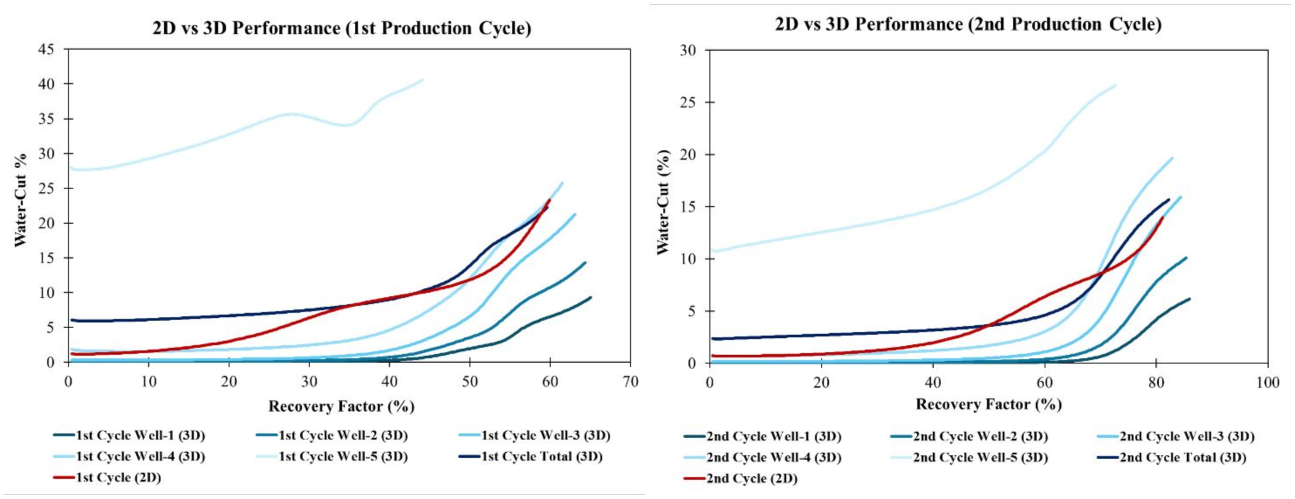

Figure S8. Omni-directional model: water-cut vs recovery factor across 2D and 3D models. Water-cut evolution during withdrawal is reported separately for each layer (Layers 1–5) and compared against the 2D baseline. The deepest layer exhibits systematically higher water cut, consistent with gravity-driven segregation, while upper layers remain closer to the 2D response. Also, gravity segregation reduces recovery in the deepest layer relative to the upper layers; however, the layer-averaged 3D performance remains close to the 2D result because the controlling X–Y heterogeneity is identical.

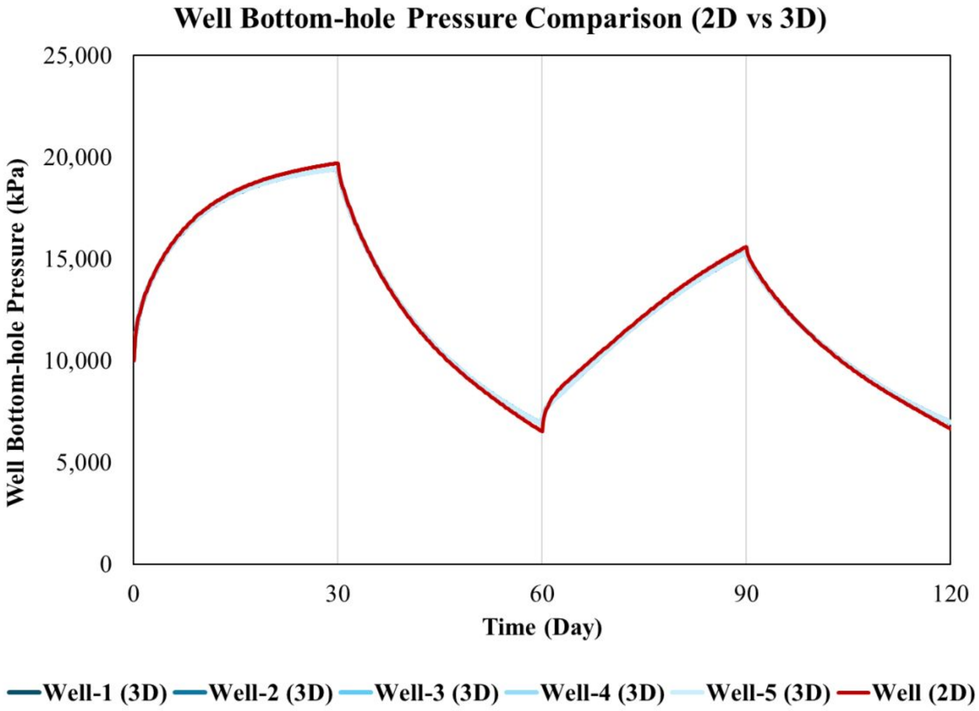

Figure S9. Omni-directional model: well bottom-hole pressure histories across 2D and 3D models. Injector/producer BHP histories comparing the 2D horizontal model (0.5 m thickness) with the 3D extension (2.5 m total thickness, five layers) in which the same X–Y permeability heterogeneity is replicated in each layer, and five horizontal completions are perforated across all layers. The pressure response remains consistent across the 3D layers and closely matches that in the 2D case, indicating that, in this configuration, pressure evolution is primarily governed by lateral connectivity and the imposed operating constraints.
